# Sputum Proteomics Reveals a Shift in Vitamin D-binding Protein and Antimicrobial Protein Axis in Tuberculosis Patients

**DOI:** 10.1038/s41598-018-37662-9

**Published:** 2019-01-31

**Authors:** Subasa C. Bishwal, Mrinal K. Das, Vinod K. Badireddy, Deepti Dabral, Aleena Das, Alok R. Mahapatra, Sukanya Sahu, Dipankar Malakar, I. Ibungo Singh, Himanghsu Mazumdar, Saurav J. Patgiri, Trinayan Deka, Wetetsho Kapfo, Kevideme Liegise, Rukuwe-u Kupa, Sanjita Debnath, Rajesh Bhowmik, Rahul Debnath, Rajendra K. Behera, Manoj G. Pillai, Pranjal Deuri, Reema Nath, K. Pewezo Khalo, W. Asoka Sing, Bhaswati Pandit, Anjan Das, Sibabrata Bhattacharya, Digambar Behera, Lahari Saikia, Vinotsole Khamo, Ranjan K. Nanda

**Affiliations:** 1 Translational Health Group, International Centre for Genetic Engineering and Biotechnology, New Delhi, India; 2 School of Life Sciences, Sambalpur University, JyotiVihar, Sambalpur, India; 3 Sciex, 121 UdyogVihar, Gurgaon, Haryana India; 4 Department of Respiratory Medicine, Regional Institute of Medical Sciences, Imphal, India; 5 Department of Microbiology, Assam Medical College and Hospital, Dibrugarh, India; 6 Healthcare Laboratory and Research Centre, Naga Hospital Authority Kohima, Nagaland, India; 7 Department of Respiratory Medicine, Agartala Government Medical College, Agartala, India; 8 National Institute of Biomedical Genomics, Kalyani, West Bengal India; 9 Department of Microbiology, Naga Hospital Authority Kohima, Nagaland, India; 10 Department of Microbiology, Agartala Government Medical College, Agartala, India; 11 Department of Pulmonary Medicine, Postgraduate Institute of Medical Education and Research, Chandigarh, India

## Abstract

Existing understanding of molecular composition of sputum and its role in tuberculosis patients is variously limited to its diagnostic potential. We sought to identify infection induced sputum proteome alteration in active/non tuberculosis patients (A/NTB) and their role in altered lung patho-physiology. Out of the study population (n = 118), sputum proteins isolated from discovery set samples (n = 20) was used for an 8-plex isobaric tag for relative and absolute concentration analysis. A minimum set of protein with at least log_2_(*ATB*/*NTB*) >±1.0 in ATB was selected as biosignature and validated in 32 samples. Predictive accuracy was calculated from area under the receiver operating characteristic curve (AUC of ROC) using a confirmatory set (n = 50) by Western blot analysis. Mass spectrometry analysis identified a set of 192 sputum proteins, out of which a signature of β-integrin, vitamin D binding protein:DBP, uteroglobin, profilin and cathelicidin antimicrobial peptide was sufficient to differentiate ATB from NTB. AUC of ROC of the biosignature was calculated to 0.75. A shift in DBP-antimicrobial peptide (AMP) axis in the lungs of tuberculosis patients is observed. The identified sputum protein signature is a promising panel to differentiate ATB from NTB groups and suggest a deregulated DBP-AMP axis in lungs of tuberculosis patients.

## Introduction

Pulmonary tuberculosis (TB) involves complex patho-physiological perturbations and present with heterogeneous complications^1^. Mucus protects the respiratory tract from the damaging action of external as well as internal particles and mucus hypersecretion is exhibited by TB patients^2^. Mucus is normally swallowed and delivered to the gastro-intestinal tract for degradation, but in respiratory disease like TB it is usually coughed out as sputum^3^. Sputum is a complex dilute aqueous solution of lipids, proteins and glycoconjugates. It also contains inhaled air particles, exogenous as well as endogenous bacterial products, antibacterial secretions, cell and plasma-derived mediators and proteins^4^.

*Mycobacterium tuberculosis* (Mtb), the causative organism of TB, resides in respiratory tract by embedding in mucous in the lumen, adhering to or internalized in respiratory epithelial cells, neutrophils and macrophages in the interstitium between cells and granuloma. Epithelial and homing immune cells in lungs produce a wide repertoire of biophysical scaffolds, host–defense molecules, cytokines, chemokines and damage-associated molecular patterns (DAMPs) to maintain near sterility throughout life^5^. Thus, to adapt and grow in these microenvironments, Mtb may alter host system for its basic survival and growth. Mtb has been reported to release molecules that compromise the host system to make it suitable for its survival^6^.

In this report, we aim to capture the Mtb infection induced sputum proteome composition alteration in drug naïve TB patients. Sputum as a matrix may provide useful additional information about the patho-physiological condition of lungs in perturbed conditions. Employing comparative quantitative proteomic profiling tools, important deregulated molecules could be identified in sputum of drug naïve active- and non-TB (A/NTB) subjects. Information thus obtained may provide vital clues as to how Mycobacteria affect the host cells, organs and cause overall changes in constituents for its own benefit.

## Materials and Methods

### Ethics statement

Following the approval of institute review boards of partnering centers (Regional Institute of Medical Sciences, Imphal (Ac/112/EC/RIMS/2005); National Institute Biomedical Genomics, Kalyani; Naga Hospital Authority, Kohima (NHAK/HLRC/RES-3/2013/64; Assam Medical College, Dibrugarh (AMC/EC/3362), and Agartala Government Medical College, Agartala(F.4(5-2)/AGMC/Academic/Project/Research/2007/Sub-II/6710-6704) and International Centre for Genetic Engineering and Biotechnology, New Delhi (ICGEB/IEC/2012/02, ICGEB/IEC/2014/06 and ICGEB/IEC/2014/07) study subjects were enrolled. All study subjects or their legal guardian provided written informed consent to participate in this study. All methods were performed in accordance with the relevant guidelines and regulations approved by the committees.

### Patient recruitment and classification

**Figure 1:**
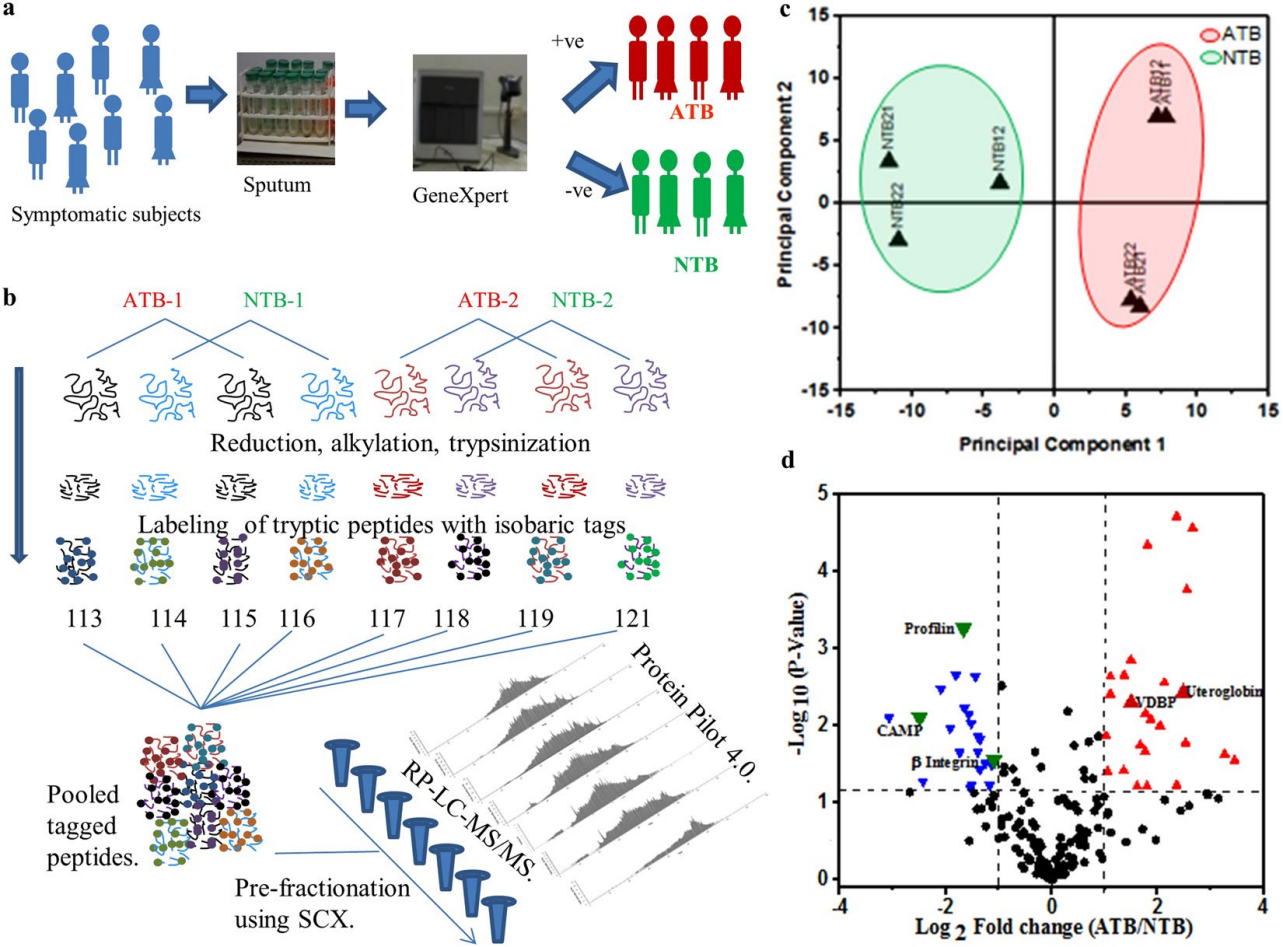
Sputum proteome of pulmonary tuberculosis patients shows deregulation. (**a**) Drug naïve tuberculosis suspects were grouped as active and non-tuberculosis patients (A/NTB) based on positive GeneXpert and microscopy findings. (**b**) An 8-plex isobaric tag for relative and absolute quantification was carried out using proteins isolated from two groups of ATB and NTB from clinical site- I. (**c**) Principal component analysis (PCA) of the protein abundance data shows two separate clusters of ATB and NTB. (**d**) The volcano plot shows the mean difference of the protein intensity plotted against the P value. The dashed lines indicate the significance cutoff values.

Adult (>15 years) subjects presenting complain to of two-weeks-old cough, chest pain, night sweat and fever to the out patients department were recruited after receiving signed informed consent. Patients with +ve HIV status were excluded. Two sputum samples with at least one early morning and one on-spot samples were collected and stored at −80 °C within 30 mins of collection. Saliva contaminated samples were excluded by monitoring presence of squamous epithelial cells by Wright’s staining. Serum samples were isolated by incubating blood samples (5 ml) for 3 hours at room temperature and supernatant was collected after centrifuging at 2,000 × *g* for 30 mins at 4 °C. WHO guidelines were followed for Ziehl-Neelsen sputum microscopy and GeneXpert analysis was carried out following recommended manufactures protocol (Cephid, USA). Subjects were classified as ATB based on positive test results for both GeneXpert and sputum microscopy, both negative results were grouped as NTB and rest were discarded (Fig. 1a). Based on the clinical presentation, the NTB subjects were representing from other disease conditions like asthma, chronic obstructive pulmonary diseases (COPD), lung cancer, pneumonia, or suffering from more than one complication. Subjects were grouped to discovery (n = 20), validation (n = 32, 8 follow up samples) and confirmatory sets (n = 50) based on the clinical sites and period of subject recruitment. Epidemiological details of all study subjects were included in Table 1. A STARD diagram with the entire patient inflow details is presented in Supplementary Fig. S1. ATB subjects (n = 8) completing two months of therapy were grouped as non responders (ATB-NR) based on positive Ziehl-Neelsen stain results and rest as responders (ATB-R). Due to remote location of clinical sites and logistic challenges, culture test of all samples could not be achieved.

**Table 1:** Epidemiological details of all study subjects used to identify important deregulated molecules in sputum of tuberculosis patients.

Subject details	Total Subjects	Discovery set		Validation set				Cytology analysis		Confirmatory mixed cohort	
				Site specific groups		Follow up set					
Study Groups	A/NTB	ATB	NTB	ATB	NTB	ATB-R	ATB-NR	ATB	NTB	ATB	NTB
No. of Subjects	**118**	10	10	16	16	4	4	4	4	26	24
Mean Age (Range) in years	**45(16–86)**	39(27–58)	35(19–50)	43(17–79)	44(16–79)	28(23–35)	49(40–60)	35(18–50)	25(16–40)	44(17–80)	55(18–86)
Gender (m/f)	**77/41**	9/1	4/6	10/6	7/9	3/1	3/1	3/1	1/3	20/6	17/7
BMI	**19.22** **±** **3.52**	20.2 ± 3.03	19.70 ± 3.1	18.8 ± 2.35	20.2 ± 4.32	18.86 ± 2.46	17.59 ± 5.5	20.86 ± 1.5	20.12 ± 4.5	19.94 ± 3.9	16.81 ± 2.9
Smoking Habit (y/n/na)	**36/65/17**	9/1/−	2/8/−	5/7/4	5/7/4	3/1/−	2/2/−	1/3/−	−/4/−	6/20/−	4/11/9
Alcoholism (y/n/na)	**18/69/31**	4/6/−	1/9/−	6/6/4	4/8/4	1/3/−	2/2/−	−/4/−	−/4−	1/17/8	−/9/15
**Clinical Symptoms**											
Cough	**118**	10	10	16	16	4	4	4	4	26	24
Expectoration	**118**	10	10	16	16	4	4	4	4	26	24
Chest pain (y/n/na)	**60/49/9**	3/7/−	2/8/−	14/2/−	14/2/−	1/3/−	4/−/−	2/2/−	2/2/−	10/15/1	7/8/9
Fever (y/n/na)	**58/24/36**	9/1/−	5/5/−	8/4/4	7/5/4	2/2/−	4/−/−	−/3/1	−/−/4	17/1/8	6/3/15
**Clinical Findings**											
AFB (+/−ve)	52/46*	10/−	−/10	16/−	−/16	4/−	4/−	4/−	−/4	24/2	−/14*
GeneXpert (+/−ve)	**64/54**	10/−	−/10	16/−	−/16	4/−	4/−	4/−	−/4	26/−	−/24
Abnormal Chest X-ray (y/n/na)	**63/12/43**	9/−/1	1/1/8	12/−/4	5/3/8	4/−/−	3/1/−	−/−/4	−/−/4	21/−/5	6/8/10
Cavity (y/n/na)	**62/11/45**	8/−/2	1/1/8	14/−/4	5/3/8	4/−/−	3/1/−	−/−/4	−/−/4	21/−/5	6/8/10

A/NTB: active-/non-tuberculosis, ATB- R/NR: tuberculosis patients responder/non-responder at 2 months of treatment; AFB: Acid fast bacilli sputum microscopy test; y/n/na: yes/no/not available; *data not available.

### Sputum cytology analysis

A subset of sputum samples (500 μl) from ATB and NTB subjects (n = 8) were fixed with equal volume of 70% ethanol specifically for cytology analysis. Smear was prepared on a glass slide, dried for 15 mins at room temperature before heat fixing and Wright’s stain was applied for 3 mins. After washing with water, slides were incubated with diluted Wrights stain (stain/phosphate buffer saline; 1:4 (v/v)) for 15 mins. After second wash with water, slides were kept at room temperature for air drying and taken for observation of presence and absence of different types of cells at 400× magnification using a compound

microscope *(Carl Zeiss)*. Epithelial cell, macrophage and neutrophil were counted from a total of 200 fields and presented in % with respect to total cell numbers.

### Sputum processing for proteomics experiment

Equal volume (1.0 ml) of stored sputum samples from five individual ATB or NTB subjects of discovery set were pooled to prepare four groups. Dithiothreitol (0.2%) was added to sputum samples and supernatant collected by centrifuging at 1,000 × *g* for 10 mins at 4 °C. Bradford reagent (Bio-Rad, USA) was used to quantify total protein with bovine serum albumin (BSA) as standard. Protein (100 µg) from each group precipitated using acetone (v/v:1/6) and dissolved in 20 µl of dissolution buffer (supplied by Sciex, USA). Recommended protocol for isobaric tags for relative and absolute quantification (iTRAQ) chemistry provided by supplier was followed to generate labeled tryptic peptides (Fig. 1b). Briefly, after reduction using dithiotheretol (50 mM) for 1 hour at 60 °C, cysteinyl residues were blocked with iodoacetamide (200 mM). Proteins were incubated with trypsin enzyme for 16 hours at 37 °C. The dried tryptic peptides were mixed with iTRAQ tags and incubated at room temperature for 2 hours. The labeled tryptic peptides were pooled together and dried at 40 °C using vacuum evaporator. A hand-held ICAT® Cartridge–cation-exchange cartridge system (AB Sciex, USA) was used to fractionate the pooled peptides and eluents of 30, 50, 80, 120, 300, 400 and 500 mM of ammonium formate solutions were collected as seven fractions. Each fraction was subjected to C18 spin column purification before mass spectrometry data acquisition.

Each peptide fraction (~32.5 μg) was dissolved in 100 μl solvent A (2% acetonitrile:ACN with 0.1% formic acid:FA) to analyze on a TripleTOF5600 (Sciex, USA) MS coupled to an Eksigent NanoLC-Ultra 2D plus system. Approximately 1.95 μg peptides (6 μl) were loaded onto a reverse phase peptide Eksigent ChromoXP trap (200 mm × 0.5 mm, 3 mm, 120 Å) column and desalted at a flow rate of 3 ml per min for 45 mins. After desalting, the peptides were separated using an Eksigent C18 column (75 mm × 15 cm, 3 mm, 100 Å) at a flow rate of 225 nl/min using a 90 min of linear gradient of 5% to 90% solvent B (98% ACN with 0.1% FA). Samples were analyzed using a nebulizing gas of 5, a curtain gas of 25, an ion spray voltage of 2600 V and a heater interface temperature of 130 °C. Information dependent acquisition (IDA) mode with a TOF MS survey scan (350–1250 m/z) with an accumulation time of 250 ms and a maximum of twelve precursor ions per cycle were selected for fragmentation. Each MS/MS spectrum was accumulated for 100 ms (100–1500 m/z) with a total cycle time of approximately 1.5 sec. Only the parent ions with a charge state from +2 to +5, threshold precursor ion intensity >120 cps, 10 sec exclusion period was selected for MS/MS fragmentation. The MS/MS spectra were acquired in high sensitivity mode with ‘adjust collision energy when using iTRAQ reagent’ settings.

### Sputum protein identification and informatics analysis

Peak lists of all seven LC-MS/MS raw data files were generated by uploading to ProteinPilot software 4.0.8085 (Revision 148085 and from Applied Biosystems SCIEX). Inbuilt ParagonTM algorithm (4.0.0.0, 148083) was employed to identify peptide sequence tags, perform data base matching for protein identification, protein grouping to remove redundant hits and comparative quantification. *Homo sapiens* protein database (UniProt/Swiss-Prot version, released March 14, 2011 with 61,113 proteins) was used for spectral library search. False discovery rates (FDR) at peptide and protein levels were calculated using the inbuilt algorithm of Protein Pilot. All protein identification was based on 95% confidence with a ProtScore of more than 1.3. Quantification results of proteins identified with minimum two unique peptides and after manual cross check, proteins with single peptide were included in the final list. Sputum proteins with at least two fold higher or lower abundance [log_2_(*ATB*/*NTB*) >±1.0] in ATB with respect to NTB were selected as important molecules. All data files were also searched against *Mycobacterium tuberculosis* (Mtb) protein database (UniProt/Swiss-Prot version, released March 5, 2011 with 4,051 proteins) to identify Mtb specific proteins. Accession number of identified proteins were used as input parameters and loaded in PANTHER (http://www.pantherdb.org/) to identify major pathway, biological processes, molecular function and protein classes. All identified and the deregulated sputum proteins in ATB patients were uploaded to STRING 10.0 (http://string-db.org/) to create a protein-protein interaction map. Proteins were represented as node and edges represent interaction with predicted interacting partners.

### Validation using Western blot analysis

Sputum protein (20 μg) of subjects from second perspective independent validation set were resolved in 12% Sodium dodecyl sulfate Poly acrylamide gel electrophoresis (SDS-PAGE) gel. The separated proteins were transferred from gel to nitrocellulose membrane using TE 77-semidry transfer unit (GE healthcare Bio-sciences, USA) at 110 volt for 90 mins. After blocking with BSA (5%) in Tris buffer saline with 0.1% tween 20 (TBST) overnight, membrane was washed and incubated with diluted (1:250) primary antibody of Vitamin D Binding protein (VDB; SC-32899, Santa Cruz Biotechnology, USA), Profilin (SC-25788), β-Integrin (SC-25714), Uteroglobin CC-10 (SC-9772), and Cathelicidin Antimicrobial Peptide (CAMP; LL-37 SC-21578) for 2 hours at room temperature. After incubation, blots were incubated for 2 hours in room temperature with anti-goat (SC-2004) or anti-rabbit IgG-HRP secondary antibody (SC-2030) at dilution of 1:5000 in TBST with BSA (1%). Blots were developed using luminal reagents (SC-2048, Santa Cruz Biotechnology, USA) and the signal was captured on Kodak film (Kodak, USA) in the dark room. Similarly serum proteins (10 μg) were loaded in 12% SDS-PAGE gel and developed following similar parameters. X-ray films were scanned and densitometry analysis was performed using Image J software. Due to close molecular weights of the marker molecules, separate immunoblots were probed in western blot analysis. Relative intensity with respect to the highest intensity band was taken for analysis. Loading quantities were monitored by running a parallel set of protein samples in a separate gel using silver stain. A third confirmatory mixed cohort (n = 50) was taken for separately calculating accuracy of the protein panel by Western blot analysis following the standardized protocol.

### Statistical analysis

In this explorative study, sample size calculation was not done. Data are shown as mean ± standard error of the mean. Protein-Pilot data were exported and used for hierarchical clustering and principal component analysis (PCA) to explore grouping patterns. Relative abundance of protein samples were used to calculate Pearson correlation coefficient between technical replicates of ATB and NTB groups. *P* values were calculated using unpaired, two-tailed Student’s t-test. P values of <0.05 were considered significant. Area under receiver operating characteristic curve (AUC or ROC) was calculated by uploading the data generated through Western analysis and generated from the confirmatory mixed cohort using MetaDisc software.

## Results

A total of 118 study subjects (male/female: 77/41; mean age 45 and in the range of 16–86 years) recruited from four clinical sites in India and collected in two different periods were included in this study (Supplementary Fig. S1). Epidemiological details of all study groups are presented in Table 1 and extended details in Supplementary Table S1. Subjects with positive results, for both GeneXpert and sputum microscopy, were grouped as active tuberculosis (ATB) and with double negative results as non tuberculosis (NTB) patients suffering from asthma, COPD, lung cancer or pneumonia (Fig. 1c). Sputum pH was found to be significantly lower in ATB than NTB subjects (Supplementary Fig. S2).

Sputum proteome isolated from biological and technical replicates were used in a comparative quantitative proteomics experiments (Fig. 1b). All mass spectrometry raw data files including peptide and protein list can be accessed from ProteomeXchange using the reference number of PXD003065 and MassIVE Accession No. MSV000079349. We identified 192 proteins the highest number of host sputum proteins reported in TB patients (Supplementary Table S2, Fig. S3). When searched against Mtb protein database, we did not identify any Mtb-specific protein. We observed high correlation in relative expression of proteins identified in technical replicates of ATB group (0.84 and 0.81), compared with NTB (0.43) (Supplementary Fig. S4). Hierarchical clustering and PCA of all identified proteins from ATB and NTB groups with their relative abundances grouped them into two separate clear clusters (Fig. 1c, Supplementary Fig. S3).

Gene ontology (GO) analysis of identified (n = 192) sputum proteins showed that majority of them execute different molecular functions such as antioxidant, binding, catalytic, enzyme regulation, structural molecular and transporter activities (Supplementary Fig. S5A). These proteins are involved in metabolic, cellular, immune system, response to stimulus, biogenesis, biological regulation, apoptotic, biological adhesion, developmental, localization and multicellular organismal process and belong to different classes (Supplementary Fig. S5B,C). Out of 192 identified proteins, 25 proteins qualified as important deregulated molecules (Fig. 1d, Supplementary Fig. S6). A protein interaction network map built using all identified proteins (Supplementary Fig. S7A) and these 25 deregulated proteins lead to three clusters (Supplementary Fig. S7B). Four proteins from these clusters (profilin, cathelicidin antimicrobial peptide: CAMP, β-integrin, vitamin D binding protein: DBP) and one important protein from airway inflammatory response (uteroglobin) as a panel classify ATB and NTB groups separately (Supplementary Figs S8 and S9). These variations in host protein abundances may be due to infection induced patho-physiological alterations in lungs. In fact, we observed similar immune cell distribution in a separate set of fixed sputum samples (ATB/NTB: 4/4) used for cytoloy analysis (Supplementary Fig. S10).

**Figure 2:**
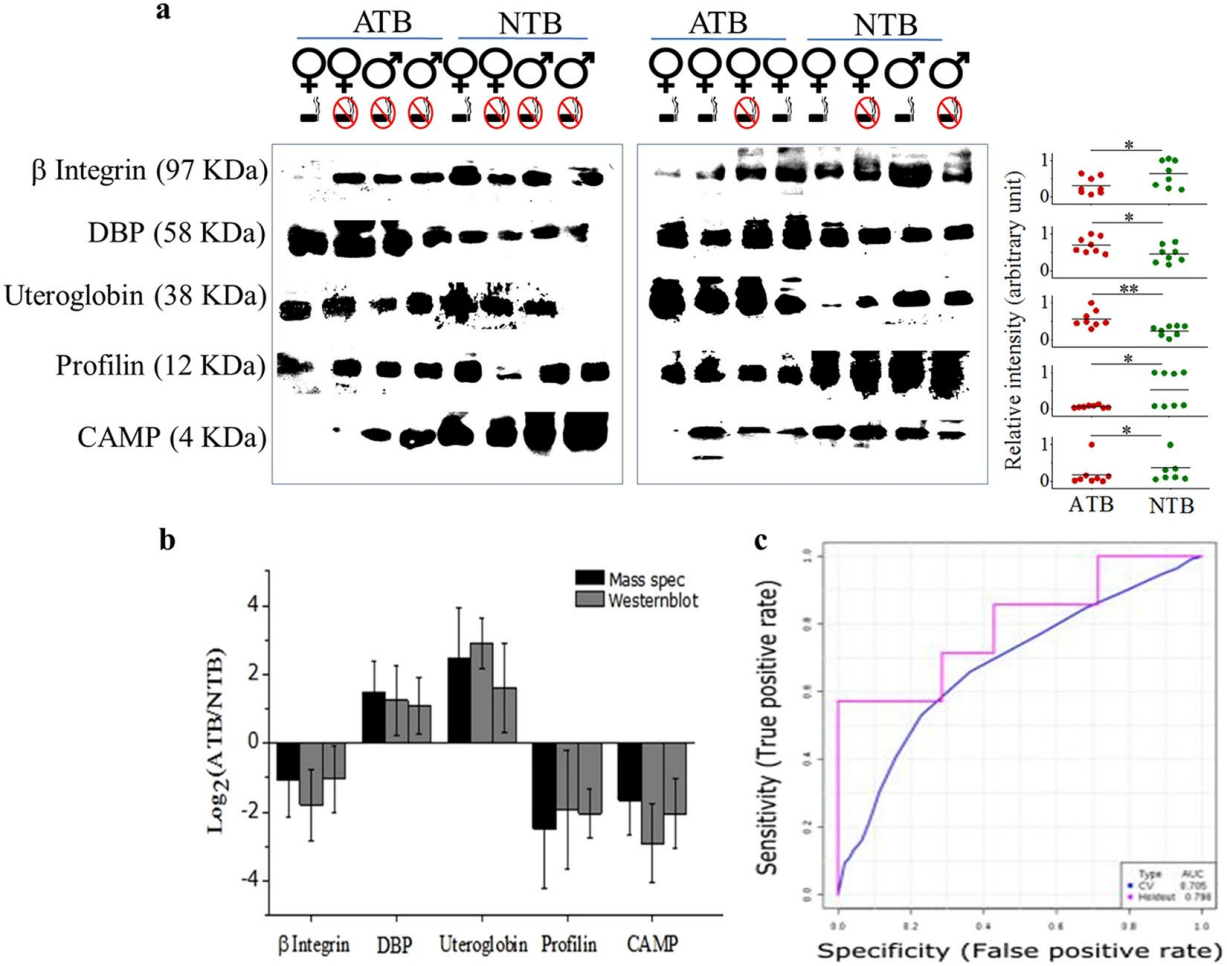
Validation of the identified important proteins in independent sample sets. (**a**,**b**) Western blot analysis of β-Integrin, vitamin D binding protein (DBP), Uteroglobin, Profilin and cathelicidin antimicrobial peptide (CAMP) with relative intensities in independent validation sample sets. (**c**) Area under the curve calculated from the confirmatory mixed cohort sample sets (n = 50). Unprocessed original scans of the western blots can be found in Supplementary Fig. S16. Data presented in means ± SD, *P < 0.05, **P < 0.01, unpaired t test. Gender and smoking status of individual subjects are presented.

The abundance of these 5 protein panel monitored in independent validation samples (n = 32) showed significant variation (Fig. 2a). In a subset using parallel biofluids (serum and sputum), we observed similar variation in sputum (Supplementary Fig. S11) and no change in serum of ATB and NTB subjects (Supplementary Fig. S12). A similar trend was also observed in the proteomics and Western blot data analysis (Fig. 2b, Table 2). In a third confirmatory mixed cohort (n = 50), AUC under ROC calculated for the same panel of five proteins was 0.75 and holdout set was 0.79 (at P > 0.05) with a sensitivity of 70% and specificity of 79% (Fig. 2c, Supplementary Fig. S13).

**Table 2:** Performance of deregulated molecules as identified from discovery and validation sets of sputum samples of active-/non-tuberculosis patients from four different clinical sites.

Important deregulated proteins	log_2_(*ATB*/*NTB*)				
	LC-MS/MS	Western blot			
	Discovery Set	Validation Sets			
β-Integrin (97 KDa)	−1.09	−1.8	−1.05	−1.003	−1.03
VDBP (58 KDa)	1.49	1.24	1.08	1.25	1.32
Uteroglobin (38 KDa)	2.48	2.90	1.60	1.09	1.40
Profilin (12 KDa)	−2.49	−1.93	−2.05	−1.05	−3.34
CAMP (4 KDa)	−1.66	−2.91	−2.05	−1.18	−1.11

**Figure 3:**
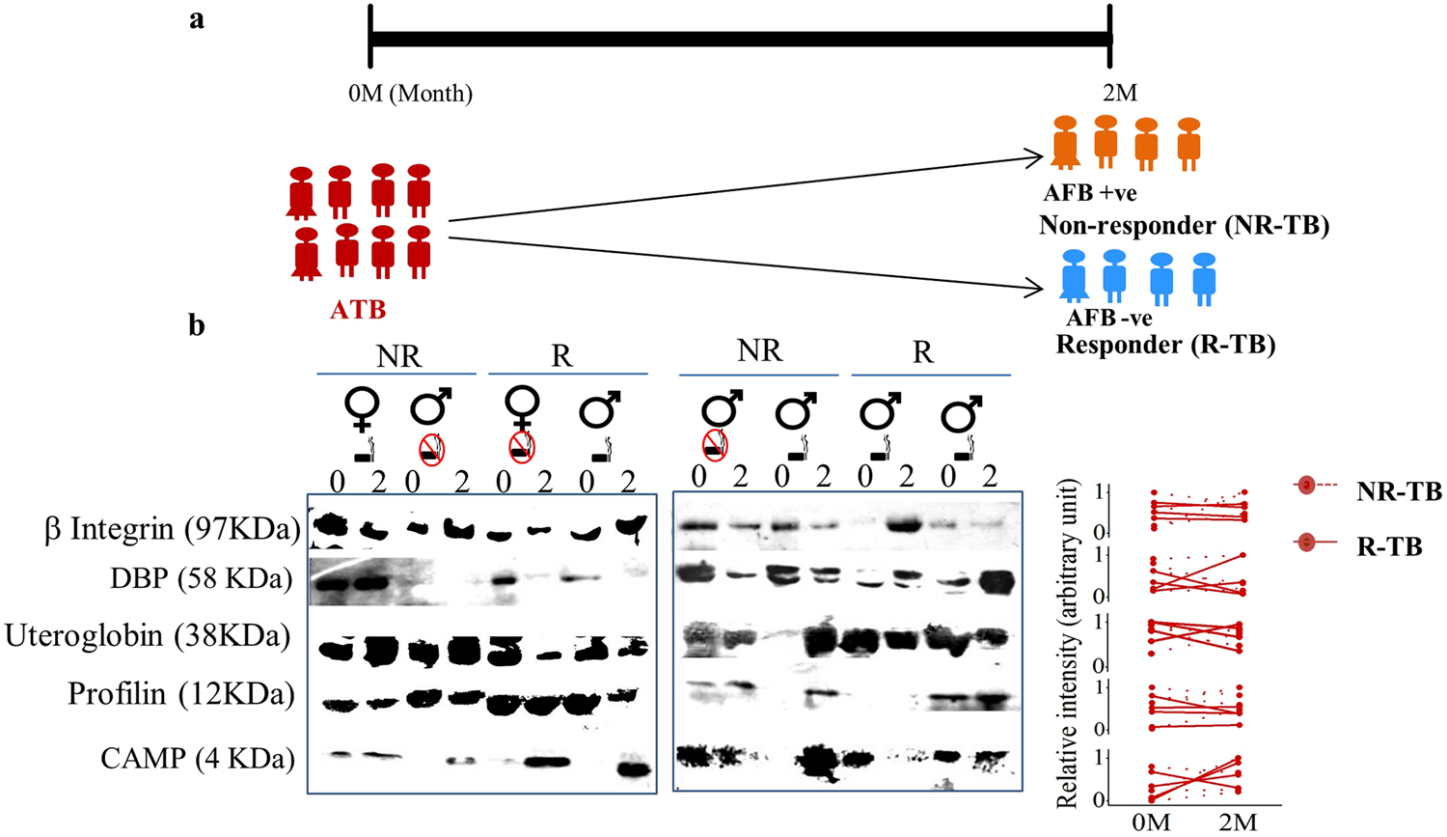
Important sputum proteins show therapy induced alteration in active tuberculosis patients that were followed longitudinally. (**a**) Tuberculosis patients were followed during receiving treatment and grouped as non-responders (NR) based on +ve microscopy results and rest as responders (R). (**b**) Individual proteins were probed using Western blot analyses and relative changes in intensities showed that sputum proteins could differentiate NR-tuberculosis (TB) from R-TB groups. Unprocessed original scans of the western blots can be found in Supplementary Fig. S17. Gender and smoking status of individual subjects are presented.

In longitudinally followed ATB subjects, we observed higher abundance of β-Integrin and uteroglobin in ATB-NR with respect to ATB-R (Fig. 3a,b). Higher abundance of CAMP was observed in ATB-R and it remained low in ATB-NR (Fig. 3b). DBP and profilin showed similar abundance between in ATB-NR and ATB-R groups (Fig. 3b).

## Discussion

To the best of our knowledge, this study is the first to apply a multiplex analysis of the sputum proteome of drug naïve ATB and NTB patients. These patients at the time of presentation need quick classification to initiate appropriate timely therapeutic intervention. We carefully selected these clinically important study groups to avoid comparing the sputum proteome of ATB subjects with induced sputum collected from healthy subjects. Similar multiplex proteomics approaches along with new tools have been used previously to identify diagnostic and prognostic markers for several other disease conditions^7,8^. Robust statistical tools have been developed to identify novel biosignature an improvement over established diagnostic procedures. We applied mass spectrometry method to identify 192 host sputum proteins which is the highest ever recorded in a previous study. The protein list covers ~28% of proteins from induced sputum of healthy subjects and 53% unique proteins^9^. An earlier report compared the sputum proteome of active tuberculosis patients with healthy controls and employed two dimensional polyacrylamide gel electrophoresis to identify deregulated molecules^10^. In fact, higher abundance of a Bacterial protein UqhC with other host proteins were reported in the sputum of tuberculosis patients compared with the healthy controls^10^. Though we do expect presence of Mtb-specific proteins in sputum of ATB patients but their low dynamic concentration may make it challenging to detect and identify them.

A 5-protein panel was identified from the discovery set that could differentiate ATB and NTB groups with moderate accuracy. Similar change in abundance was observed in prospectively collected independent second validation and third confirmatory sample sets. Each protein components of this signature represent different molecular pathways recognized in the pathophysiology of tuberculosis patients and as a signature are more conclusive than when used individually. Additionally, our findings highlight the importance of tissue-specific proteomics study to understand perturbed conditions induced by infection rather focusing on the alterations in systemic circulation. More importantly, such approach has lead to the identification of a panel of proteins capable of modestly differentiating ATB patients from NTB individuals.

**Figure 4:**
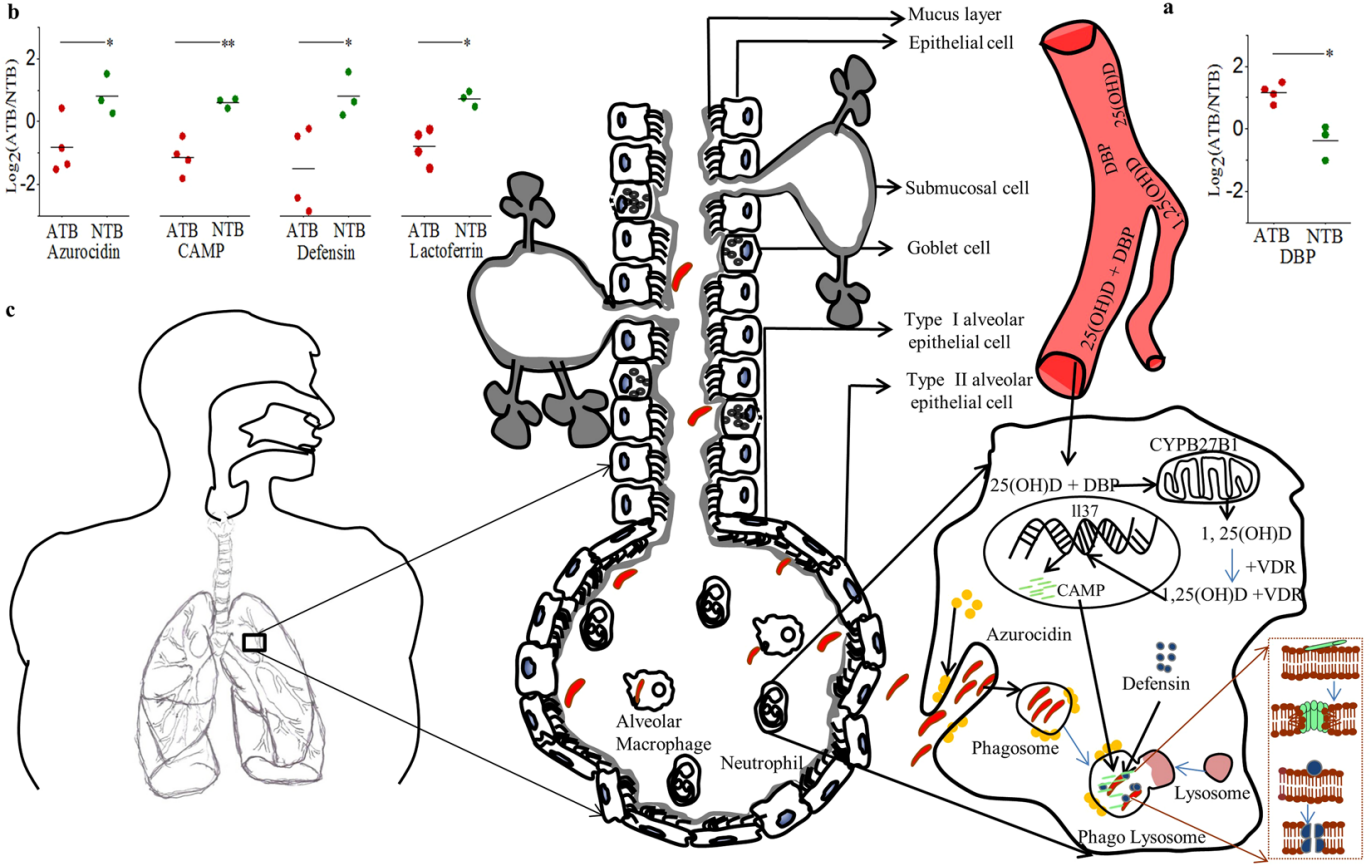
Sputum proteome analysis show tissue specific pathophysiology in tuberculosis patients which is absent in systemic circulation. Low vitamin D in circulation leads to higher DBP (**a**) in sputum to maximize the effectiveness of antimicrobial protein production. Whereas lower levels of AMP i.e. azurocidin, CAMP, defensin alpha 3 and lactoferrin are observed in sputum proteome of TB patients (**b**). (**c**) Altered DBP and AMP axis is favoring pathogen survival in the lungs of tuberculosis patients. Data are mean ± SD, *P < 0.05, **P < 0.01, unpaired t-test.

While hyper-secretion of mucus from submucosal cells is an evolutionarily conserved process developed by host to improve bacterial clearance from infected lungs, bacteria uses it for transmission (Fig. 4). After the pathogens reach the lung surface, homing of immune cells leads to 10 to 80% increase in neutrophil numbers within hours in the mucus^11^. Earlier report in asthma patients showed that spontaneous and induced sputum showed similar cellular content but were not equivalent^12^. Cytology analysis showed significantly high neutrophil percentage in sputum of both ATB and NTB subjects. In fact, we also observed higher abundance of complement C3 [log_2_(*ATB*/*NTB*) >1.86] and C4-B [log_2_(*ATB*/*NTB*) >1.97] proteins in the sputum samples of ATB subjects from NTB groups. Our observation corroborates earlier findings on how VDP enhance the neutrophil chemotactic activity of complement-derived peptides^13^. In these homing immune cells like neutrophils and macrophages, the DBP and 25(OH)D complex gets internalized to further hydroxylate to 1,25(OH)2D in mitochondria by over expression of CYPB27B1. As circulating 25(OH)D in ATB is insufficient, host accumulates DBP at the infected site to maximally utilize the available 25(OH)D. Vitamin D metabolites bind primarily to DBP but ~20% are transported by albumin^14^. We find similar sputum albumin abundance in both ATB and NTB groups and so its contribution in transporting vitamin D metabolites remains similar in the affected organ (Supplementary Fig. S14). And DBP showed a characteristic higher abundance in affected organ of ATB groups and remain unchanged in systemic circulation (Fig. 4a).

Mtb infection leads to differential expression of Integrin in T cells leading to lymphocyte trafficking to lungs^15^. We observed lower abundance of sputum profilin and may be hampering actin polymerization in affected tissue. Higher abundance of sputum uteroglobin is reported in asthma patients and these molecules play an important immunosuppressive and anti-inflammatory role in the lung^16^. Higher amount of CAMP in responders may explains higher bacterial clearance and it may be interesting to explore further the contribution of β-Integrin, profilin and CAMP in differentiating responders and non responding ATB groups.

Cytoplasmic vitamin D receptor (VDR) forms a complex with 1,25(OH)2D and acts as an important ligand-activated transcription factor that bind to regulatory regions of target genes^17^. The complex activates expression of AMP production and affects neutrophil chemo-tactic activity of complement proteins^13,14,18,19^. However, we observed lower abundance of AMP like azurocidin (log_2_(*ATB*/*NTB*) >−1.51 and p-value 0.05), CAMP (log_2_(*ATB*/*NTB*) >−1.16 and p-value 0.0005), defensin alpha 3 (log_2_(*ATB*/*NTB*) >−2.43 and p-value 0.05) and lactoferrin (log_2_(*ATB*/*NTB*) >−1.44 and p-value 0.002) in ATB (Fig. 4b). These proteins also work as chemo-attractant and activator of immune cells. Low abundance of AMP in ATB patients leads to compromise the leukocyte activation and decreased killing of internalized Mycobacteria present in phagolysosome^19^. Defensin may induce permeable lesions in Mtb cell wall to kill directly. Lower sputum pH in ATB patients, as observed in this study, may also compromise the effectiveness of the available AMPs^20^.

Corroborating earlier findings^21^, we also observed lower expression of sputum Matrix metallopeptidase-9 (log_2_(*ATB*/*NTB*) >−1.65 and p-value < 0.005) and neutrophil elastase (NE: log_2_(*ATB*/*NTB*) >−1.13 and p-value 0.03) in ATB (Supplementary Fig. S15). Reduction in NE, compromises protease production and may directly influence clearance of Mycobacteria. It is well known that NE plays a critical role in the innate immunity and inflammation, particularly in the process of neutrophil recruitment and mucin gene expression^22^. Mucin-protein interactions dynamically control the critical innate immune functions of the airway mucosal barrier including physical, biological (antimicrobial and antioxidant) functions^21^. Lactoferrin plays critical role in biofilm production and in ATB patients we observed lower abundance of it. Our result establishes that the antimicrobial and antiprotease protective screens in the lungs are compromised in TB patients. Interestingly, induction of the CAMP gene by 25(OH)D is not evolutionary conserved, as mice, rats and dogs lack a VDRE in the promoter of the CRAMP gene and thus making it difficult to study CAMP induction by 25(OH)D in animal models systems (Fig. 4c)^23–27^. Therefore, clinical proteomics studies using matrix obtained directly from patients and affected organs play a critical role in generating such evidences.

In conclusion, we report here a comparative quantitative sputum proteomics study to identify biomarkers and to understand the perturbed patho-physiology in lungs of drug naïve freshly diagnosed active- and non-tuberculosis patients. We identified a biosignature consist of 5 host sputum proteins that could differentiate ATB from NTB with modest accuracy. Our results reveal hitherto unobserved and unexpected shift in DBP and AMP axis in lungs of tuberculosis patient, a process that is likely to favor the bacteria in surviving within the host while having a negative impact on the host. Finally, our data provide evidence of a novel paradigm for understanding the altered patho-physiological system in lungs of tuberculosis patients. Such data will not only allow rapid and confident identification of ATB individuals and with more detailed follow up studies identify novel targets to develop host-directed tuberculosis therapeutics.

## Supplementary information

Supporting information
