# Supplementary material for: Sputum Proteomics Reveals a Shift in Vitamin D-binding Protein and Antimicrobial Protein Axis in Tuberculosis Patients
Source: Sci Rep. 2019 Jan 31;9:1036. doi: 10.1038/s41598-018-37662-9 (PMC6355791; doi:10.1038/s41598-018-37662-9)
Supplement: Supplementary file 1 — Supporting information [file 41598_2018_37662_MOESM1_ESM.docx]

**“Supplementary Information for Publication”**

**Sputum Proteomics Reveals a Shift in Vitamin D-binding Protein and Antimicrobial Protein Axis in Tuberculosis Patients.**

Subasa C. Bishwal, Mrinal K. Das, Vinod K. Badireddy, Deepti Dabral, Aleena Das, Alok R. Mahapatra, Sukanya Sahu, Dipankar Malakar, I. Ibungo Singh, Himanghsu Mazumdar, Saurav J. Patgiri, Trinayan Deka, Wetetsho Kapfo, Kevideme Liegise, Rukuwe-u Kupa, Sanjita Debnath, Rajesh Bhowmik, Rahul Debnath, Rajendra K. Behera, Manoj G. Pillai, Pranjal Deuri5, Reema Nath, K. Pewezo Khalo, W. Asoka Sing, Bhaswati Pandit, Anjan Das, Sibabrata Bhattacharya, Digambar Behera, Lahari Saikia, Vinotsole Khamo, and Ranjan K. Nanda*

*Correspondence author. Email: ranjan@icgeb.res.in

This file includes:

Figs. S1 to S20

Table S1 to S2

**Fig S-1**

**
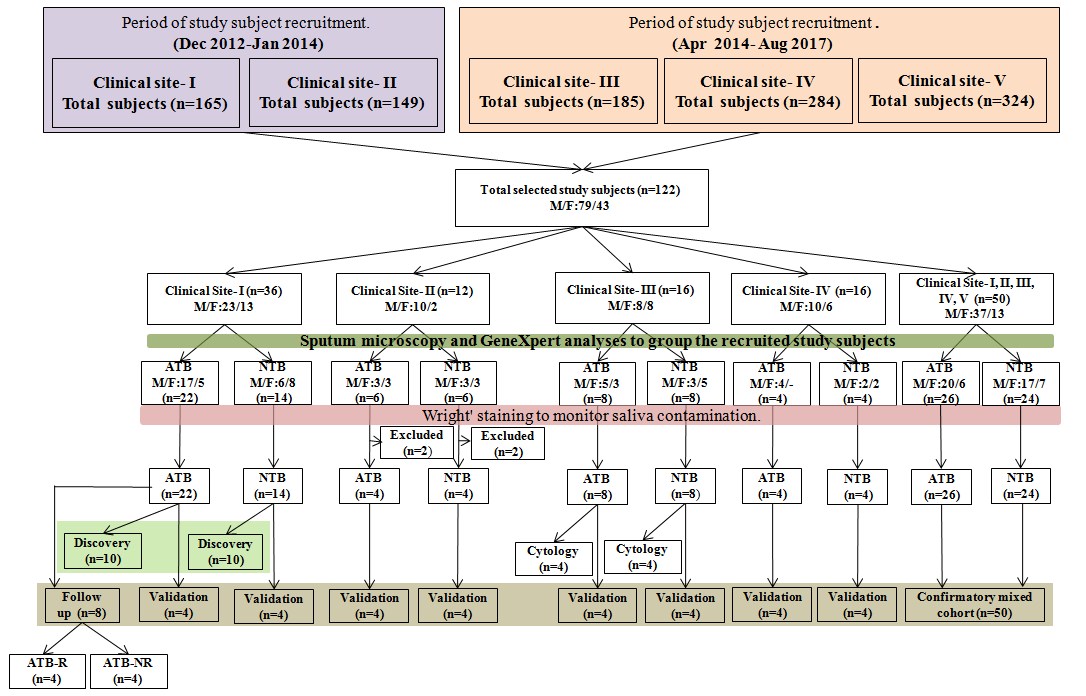
**

**Fig S-1: STARD diagram explaining study subject enrollment from multiple clinical sites, selection and grouping.** Subjects were enrolled from four clinical sites in two periods. Subjects were grouped to active and non-tuberculosis cases (A/NTB) based on +ve or -ve both sputum microscopy and GeneXpert test results. For longitudinal study, eight ATB subjects were followed and the sputum samples were collected at an interval of two months till the completion of the therapy. Based on sputum microscopy test results (+ve or -ve subjects were grouped as non-responders or responders (NR-/R-ATB). M/F:Male/Female.

**Fig S-2**

**A B**





**Fig S-2: Lower sputum pH of active tuberculosis patients was observed than non tuberculosis patients.** At the time of case presentation, sputum pH of ATB subjects showed statistical difference between ATB and NTB subjects used in discovery (A) and (B) validation Set. Horizontal line represents group mean pH values. *P < 0.05, unpaired test.

**Fig S-3**

**Fig S-3: Identified host sputum proteins, from biological and technical replicates of active and non tuberculosis groups (A/NTB), shows group specificity**. A heat map generated using the relative protein abundance of 192 proteins identified from a comparative proteomics experiment from ATB and NTB groups.

Fig S-4

A B

C

**Fig S-4:** **Biological replicates of active tuberculosis subjects show high correlation in their protein abundance.** Comparison of relative abundance of sputum proteome isolated from active and non-tuberculosis patients (A/NTB). Scatter plot showing relative protein abundance data of technical replicates of groups used in discovery set ATB 11-ATB 12/NTB 11 (A), ATB 21 - ATB 22/NTB 11 (B) and NTB 21 - NTB 22/NTB 11 (C). Linear regression analysis was carried to calculate correlation (R^2^) values between groups.

**Fig S-5**

**Figure S-5**: **Gene Ontology (GO) analysis of identified sputum proteins of active and non-tuberculosis patients (A/NTB)**. Panther analysis of the complete identified sputum proteins (192) presents diverse (A) Molecular Function, (B) Biological processes and (C) Protein Classes.

**Fig S-6**


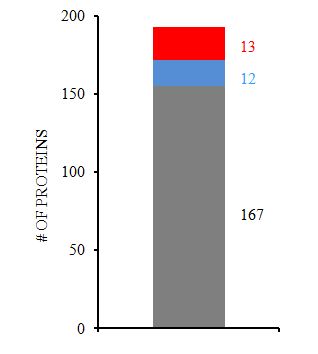


**Fig S-6:** **A small subset of sputum proteome show deregulation in active tuberculosis patients (ATB) with respect to non tuberculosis patients (NTB) with majority remain similar in their abundance.** A total of 192 host sputum proteins were identified and 147 remain unaltered (grey), and 13 showed up regulation and rest 12 were down regulated (blue). Identified proteins with at least two fold higher or lower abundance [log_2_(*ATB*/*NTB*)>±1.0] in ATB with respect to NTB were selected as important molecules.

**Fig S-7**

**A B**


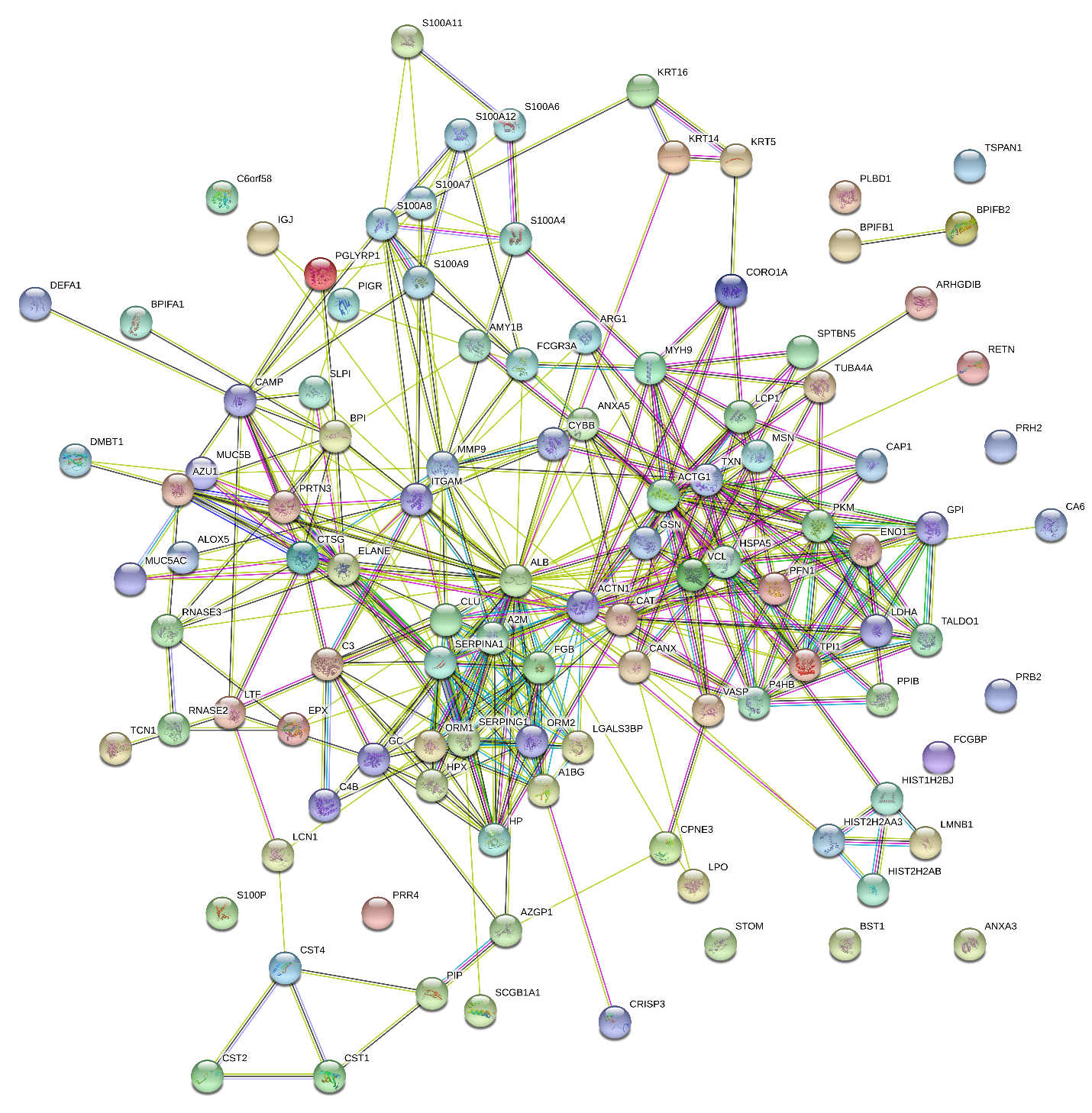


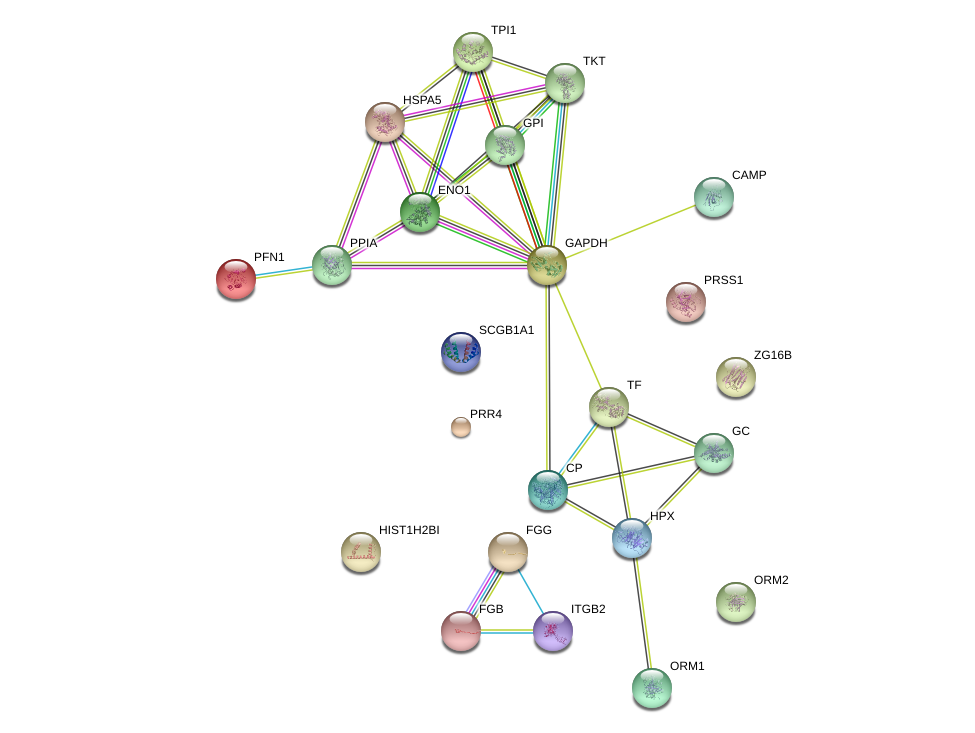


**Fig S-7: Protein-protein network of all identified (A) and deregulated proteins (B) created using the STRING database. A.** Reported functional linkage of 192 proteins with 108 nodes, 385 edges, average node degree 7.13 and clustering coefficient 0.536 with PPI enrichment p-value:< 1.0e-16. Enrichment indicated that the proteins have more interactions among themselves and at least partially biologically connected as a group. **B. Reported functional linkage of 25 important deregulated proteins** with 23 nodes, 31 edges, average node degree 2.7 and clustering coefficient 0.87. Medium confidence (STRING score=0.4) was set for network analysis. Seven different line color namely; green, red, blue, black, purple, light blue, yellow was used for evidence for association, neighborhood association, gene function, co-occurrence, co-expression, experimental, data base and text mining respectively. The clusters are marked in the red circle.

**Fig S-8**

**Fig S-8:**  A minimum of set of five proteins could differentiate ATB from NTB group and hence selected as important molecules for further validation.

**Fig S-9**

**A. Cathelicidin antimicrobial protein (CAMP)**

MKTQRDGHSLGRWSLVLLLLGLVMPLAIIAQVLSYKEAVLR**AIDGINQRSSDANLYR**LLDLDPRPTMDGDPDTPKPVSFTVKETVCPR**TTQQSPEDCDFKKDGLVK**R**CMGTVTLNQARGSFDISCDKDNKRFALLGDFFRK**SKEKIGKEFKRIVQRIKDFLRNLVPRTES

FALLGDFFR (0.0000, 1388.7761, 2, iTRAQ8plex@N-term)

RFALLGDFFR (0.0009, 1544.8781, 3, iTRAQ8plex@N-term)

CMGTVTLNQAR (-0.0004, 1542.7618, 3, iTRAQ8plex@N-term, Methylthio(C)@1)

AIDGINQR (-0.0030, 1189.6693, 2, iTRAQ8plex@N-term)

**B. Vitamin D binding protein (VDB)**

MKRVLVLLLAVAFGHALERGRDYEKNKVCKEFSHLGKEDFTSLSLVLYSRKFPSGTFEQVSQLVKEVVSLTEACCAEGADPDCYDTR**TSALSAK**SCESNSPFPVHPGTAECCTKEGLER**KLCMAALK**HQPQEFPT**YVEPTNDEICEAFR**KDPKEYANQFMWEYSTNYGQAPLSLLVSYTKSYLSMVGSCCTSASPTVCFLKERLQLK**HLSLLTTLSNR**VCSQYAAYGEKKSRLSNLIKLAQK**VPTADLEDVLPLAEDITNILSK**CCESASEDCMAK**ELPEHTVK**LCDNLSTK

Fig S9 cont..

NSKFEDCCQEKTAMDVFVCTYFMPA**AQLPELPDVELPTNK**DVCDPGNT**KVMDKYTFELSRRTHLPEVFLSK**VLEPTLKSLGECCDVEDSTTCFNAKGPLLKKELSSFIDKGQELCADYSENTFTEYKKKLAERLKAKLPDATPKELAKLVNKRSDFASNCCSINSPPLYCDSEIDAELKNIL

ADLEDVLPLAEDITNILSK (-0.0013, 2676.5034, 3, iTRAQ8plex@N-term, iTRAQ8plex(K)@19)

VPTADLEDVLPLAEDITNILSK (0.0010, 2973.6746, 4, iTRAQ8plex@N-term, iTRAQ8plex(K)@22)

RTHLPEVFLSK (-0.0015, 1934.1549, 4, iTRAQ8plex@N-term, iTRAQ8plex(K)@11)

**C. β Integrin**

GTRLSLGCVLSQECTKFKVSSCRECIESGPGCTWCQKLNFTGPGDPDSIRCDTRPQLLMRGCAADDIMDPT**SLAETQEDHNGGQK**QLSPQKVTLYLRPGQAAAFNVTFRRAK**GYPIDLYYLMDLSYSMLDDLR**NVKKLGGDLLR**ALNEITESGR**IGFGSFVDKTVLPFVNTHPDKLRNPCPNKEK**ECQPPFAFRHVLK**LTNNSNQFQTEVGKQLISGNLDAPEGGLDAMMQVAACPEEIGWRNVTRLLVFATDDGFHFAGDGKLGAILTPNDGRCHLEDNLYKRSNEFDYPSVGQLAHKLAENNIQPIFAVTSRMVKTYEKLTEIIPK**SAVGELSEDSSNVVHLIK**NAYNKLSSRVFLDHNALPDTLKVTYDSFCSNGVTHRNQPR**GDCDGVQINVPITFQVKVTATECIQEQSFVIR**ALGFTDIVTVQVLPQCECRCRDQSRDRSLCHGKGFLECGICRCDTGYIGKNCECQTQGRSSQELEGSCRKDNNSIICSGLGDCVCGQCLCHTSDVPGKLIYGQYCECDTINCERYNGQVCGGPGRGLCFCGKCRCHPGFEGSACQCERTTEGCLNPRRVECSGRGRCRCNVCECHSGYQLPLCQECPGCPSPCGKYISCAECLKFEKGPFGKNCSAACPGLQLSNNPVKGRTCKERDSEGCWVAYTLEQQDGMDRYLIYVDESRECVAGPNIAAIVGGTVAGIVLIGILLLVIWKALIHLSDLREYRRFEKEKLKSQWNNDNPLFKSATTTVMNPKFAES

Fig S9 cont..

GYPIDLYYLMDLSYSMLDDLR (0.0072, 2859.4089, 3, iTRAQ8plex@N-term)

VTATECIQEQSFVIR (-0.0036, 2073.0505, 3, iTRAQ8plex@N-term, Methylthio(C)@6)

DLYYLMDLSYSMLDDLR (0.0062, 2429.1863, 3, iTRAQ8plex@N-term)

**D. Profilin**

MAGWNAYIDNLMADGTCQDA**AIVGYKDSPSVWAAVPGKTFVNITPAEVGVLVGK**DR**SSFYVNGLTLGGQK**CSVIR**DSLLQDGEFSMDLR**TK**STGGAPTFNVTVTK**TDK**TLVLLMGKEGVHGGLINKKCYEMASHLR**RSQY

DSLLQDGEFSMDLR (-0.0002, 1928.9454, 3, iTRAQ8plex@N-term)

DSPSVWAAVPGK (-0.0027, 1821.0220, 3, iTRAQ8plex@N-term, iTRAQ8plex(K)@12)

Fig S9 cont..

KDSPSVWAAVPGK (-0.0024, 2253.3225, 4, iTRAQ8plex@N-term, iTRAQ8plex(K)@1, iTRAQ8plex(K)@13)

NITPAEVGVLVGK (-0.0016, 1904.1541, 3, iTRAQ8plex@N-term, iTRAQ8plex(K)@13)

SSFYVNGLTLGGQK (-0.0020, 2079.1440, 4, iTRAQ8plex@N-term, Deamidated(N)@6, iTRAQ8plex(K)@14)

TFVNITPAEVGVLVGK (-0.0016, 2251.3386, 4, iTRAQ8plex@N-term, iTRAQ8plex(K)@16)

CYEMASHLR (-0.0035, 1458.6691, 3, iTRAQ8plex@N-term, Methylthio(C)@1)

TLVLLMGK (-0.0041, 1481.9425, 3, iTRAQ8plex@N-term iTRAQ8plex(K)@8)

**E. Uteroglobin**

MKLAVTLTLVTLALCCSSASA**EICPSFQRVIETLLMDTPSSYEAAMELFSPDQDMREAGAQLKKLVDTLPQKPR**ESIIKLMEKIAQSSLCN

Fig S9 cont..

AMELFSPDQDMR (-0.0009, 1742.8264, 2, iTRAQ8plex@N-term)

IETLLMDTPSSYEAAMELFSPDQDMR (0.0006, 3309.5400, 4, iTRAQ8plex@N-term, Oxidation(M)@6)

KLVDTLPQKPR (-0.0044, 2206.3884, 4, iTRAQ8plex@N-term, iTRAQ8plex(K)@1, iTRAQ8plex(K)@9)

LLMDTPSSYEAAMELFSPDQDMR (0.0036, 2950.3740, 3, iTRAQ8plex@N-term)

LVDTLPQKPR (0.0004, 1774.0929, 3, iTRAQ8plex@N-term, iTRAQ8plex(K)@8)

MDTPSSYEAAMELFSPDQDMR (0.0028, 2724.2053, 3, iTRAQ8plex@N-term)

Fig S9 cont..

SYEAAMELFSPDQDMR (-0.0031, 2192.9995, 3, iTRAQ8plex@N-term)

MELFSPDQDMR (-0.0015, 1671.7888, 3, iTRAQ8plex@N term)

**Fig S-9:** Identified important sputum proteins (**A. Cathelicidin antimicrobial protein**; B. **Vitamin D binding protein**; C. **β Integrin**; D. **Profilin**; and E. Uteroglobin)from their tryptic peptide products (MS/MS spectra) labeled with isobaric tags, modifications that show significant deregulation in active- from non-tuberculosis patients (A/NTB). Peptide details like delta mass, precursor ion mass, change and modification details are presented.

**Fig S-10**


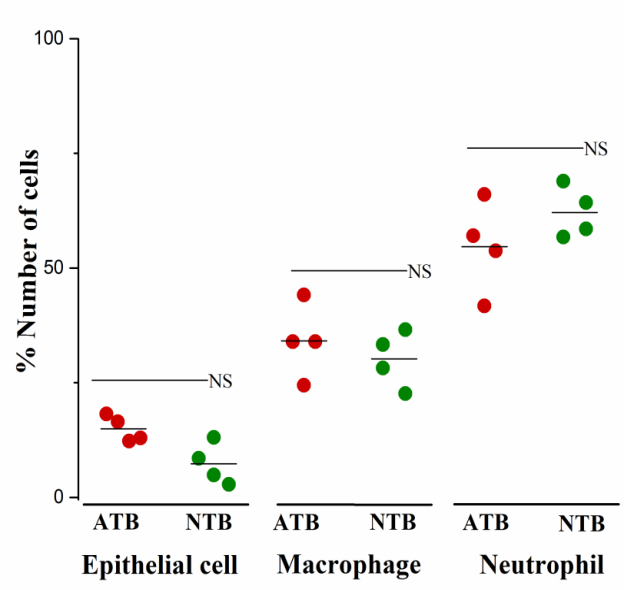


**Fig S-10: Differential cell count in sputum of active and non tuberculosis (ATB and NTB) subjects showed high degree of similarity.** Smears of sputum samples fixed in 70% ethanol from both study groups (active/non-tuberculosis, A/NTB: n=4) were stained with Wright’s stain. Different cells were counted from 20 different fields at 400 × magnification under a light microscope. Total percentages of each cell types (epithelial cell, macrophage and neutrophil) were calculated. The difference between two groups was tested using t-test and P≤0.05 was considering as significant. Horizontal bar indicates population mean value. NS: not significant at P<0.05, unpaired t test.

**Fig S-11**

**Fig S-11:** Western blot analysis of β Integrin, vitamin D binding protein (VDP), Uteroglobin, Profilin and cathelicidin antimicrobial peptide (CAMP) with relative intensities in independent validation sample sets. Unprocessed original scans of the western blots can be found in Supplementary fig. S18.

**Fig S-12**

**
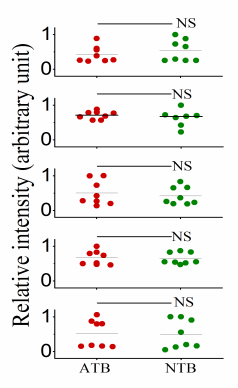
**

**Fig S-12: Monitoring the important proteins in serum of active/non-tuberculosis patients (A/NTB), recruited from clinical site- III and- IV, do not show significance change in their abundance.** (A) Western blot analysis of β Integrin, VDBP, Uteroglobin and CAMP of ATB and NTB patients. (B) Relative intensity of western blot information of individual serum samples. Unprocessed original scans of the western blots can be found in Supplementary fig. S19. Horizontal bar indicates population mean value. NS: not significant at P<0.05, unpaired t-test.

.

**Fig S-13**

**Fig S-13:** Western blot analysis of β Integrin, vitamin D binding protein (VDP), Uteroglobin, Profilin and cathelicidin antimicrobial peptide (CAMP) used for calculating area under receiver operating characteristic curve (AUC of ROC) in independent blinded confirmatory sample set (n=50). Unprocessed original scans of the western blots can be found in Supplementary fig. S20.

**Fig S-14**

**
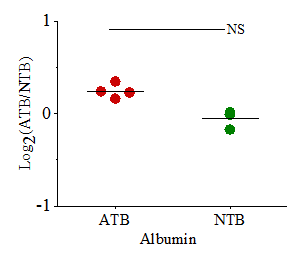
**

**Fig S-14: Relative sputum albumin abundance, as determined from mass spectrometry data, in active- and non-tuberculosis patients (A/NTB) showed similar abundance.** Horizontal bar indicates population mean relative abundance value. NS: not significant at P<0.05, unpaired t test.

**Fig S-15**

**
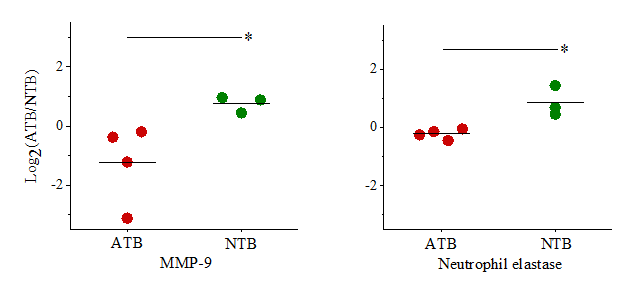
**

**Fig S-15: Mass spectrometry data showed lower abundance of Matrix metallopeptidase 9 (MMP- 9) and Neutrophil elastase in sputum of active- and non-tuberculosis patients.** *P < 0.05, unpaired t test. Horizontal line represents group mean fold change value. *P < 0.05, unpaired test.

**Fig S-16**

| 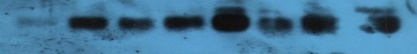 | 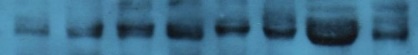 |
| --- | --- |
| 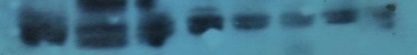 | 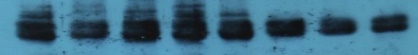 |
| 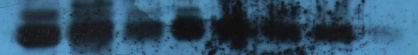 | 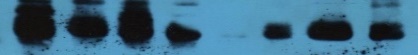 |
| 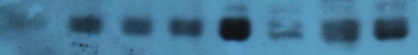 | 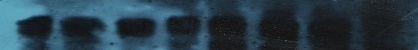 |
| 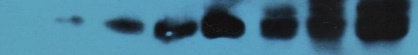 | 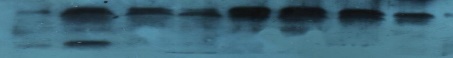 |

β Integrin (97 KDa)

DBP (58 KDa)

Uteroglobin (38 KDa)

Profilin (12 KDa)

CAMP (4 KDa)

\

**Fig S-16:** Original Western images used for preparing Figure 2a.

**Fig S-17**

| 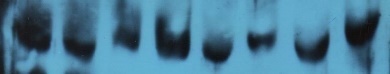 | 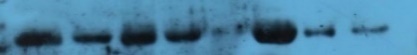 |
| --- | --- |
| 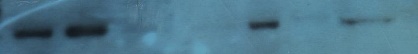 | 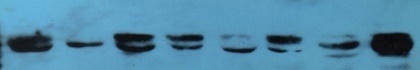 |
| 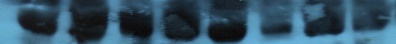 | 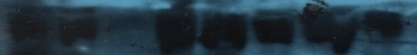 |
| 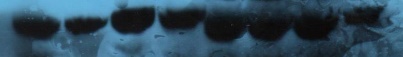 | 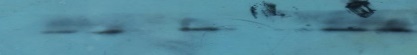 |
| 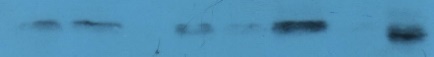 | 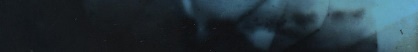 |

β Integrin (97 KDa)

DBP (58 KDa)

Uteroglobin (38 KDa)

Profilin (12 KDa)

CAMP (4 KDa)

**Fig S-17:** Original Western images used for preparing Figure 3b.

**Fig S-18**

| 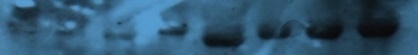 | 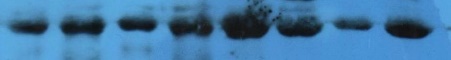 |
| --- | --- |
| 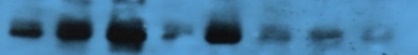 | 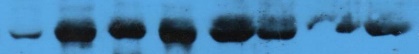 |
| 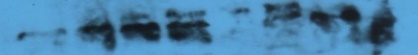 | 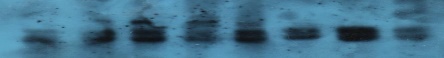 |
| 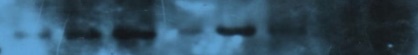 | 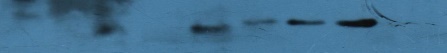 |
| 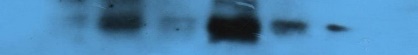 | 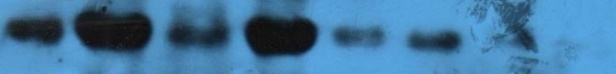 |

β Integrin (97 KDa)

DBP (58 KDa)

Uteroglobin (38 KDa)

Profilin (12 KDa)

CAMP (4 KDa)

**Fig S-18:** Original Western images used for preparing Supplementary fig. S11.

**Fig S-19**

| 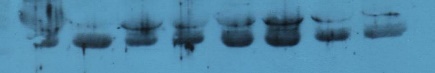 | 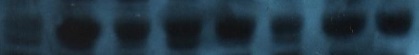 |
| --- | --- |
| 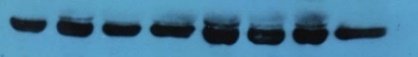 | 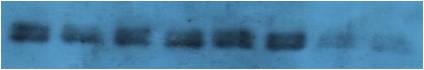 |
| 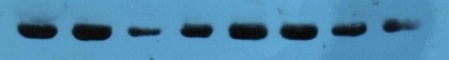 | 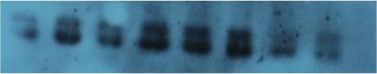 |
| 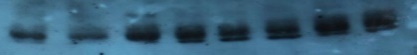 | 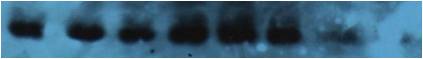 |
| 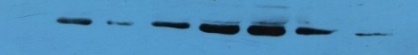 | 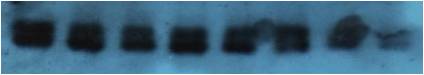 |

β Integrin (97 KDa)

DBP (58 KDa)

Uteroglobin (38 KDa)

Profilin (12 KDa)

CAMP (4 KDa)

**Fig S-19:** Original Western images used for preparing Supplementary fig. S12.

**Fig S-20**

| 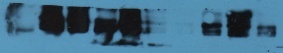 | 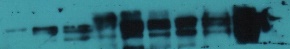 | 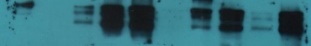 |
| --- | --- | --- |
| 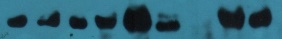 | 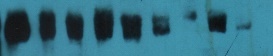 | 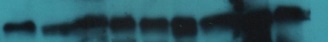 |
| 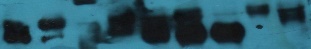 | 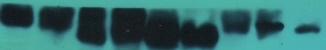 | 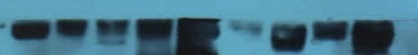 |
| 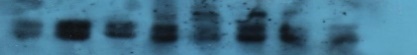 | 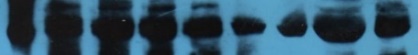 | 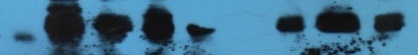 |
| 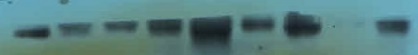 | 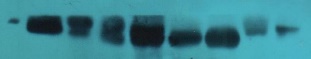 | 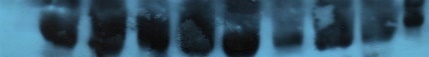 |

β Integrin (97 KDa)

DBP (58 KDa)

Uteroglobin (38 KDa)

Profilin (12 KDa)

CAMP (4 KDa)

β Integrin (97 KDa)

DBP (58 KDa)

Uteroglobin (38 KDa)

Profilin (12 KDa)

CAMP (4 KDa)

| 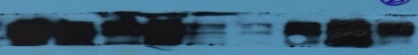 | 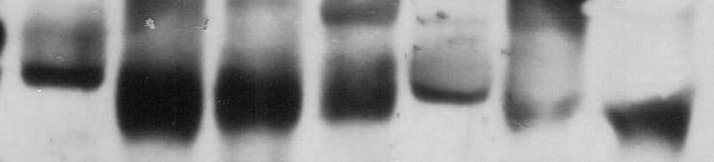 | 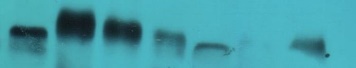 |
| --- | --- | --- |
| 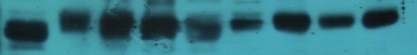 | 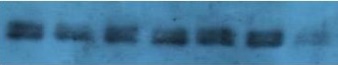 | 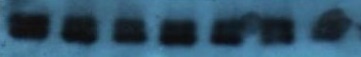 |
| 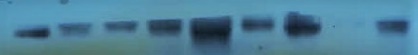 | 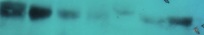 | 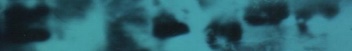 |
| 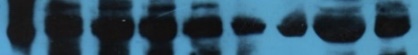 | 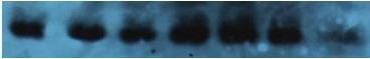 | 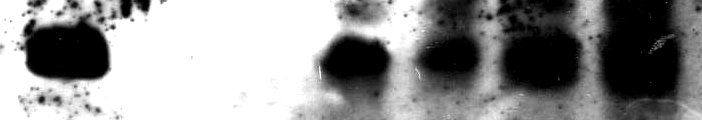 |
| 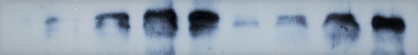 | 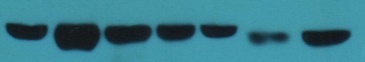 | 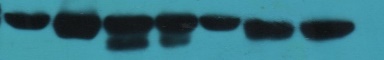 |

**Fig S-20:** Original Western images used for preparing Supplementary fig. S13.

Table S-1: Extended epidemiological details of the subjects used in this study. **(Page S-27 to S-31)**

| **Study sets** | **Clinical Sites** | **Study groupes** | **Subject code** | **Age (in Years)** | **Gender (M/F)** | **Height (m)** | **Weight (kg)** | **BMI (kg/m^2^)** | **Cough (Y/N)** | **Expectoration (Y/N)** | **Hemoptysis (Y/N)** | **Chest pain (Y/N)** | **Fever (Y/N/NA)** | **Smoking habit (Y/N/NA)** | **Alcoholism (Y/N/NA)** | **Sputum microscopy test** | | **Abnormal Chest X- ray (Y/N/NA)** | **Cavity (Y/N/NA)** | **GeneXpert esult at presentation** | **Active/non tuberculosis** | **Sputum microscopy result at 2M** | |  |  |  |
| --- | --- | --- | --- | --- | --- | --- | --- | --- | --- | --- | --- | --- | --- | --- | --- | --- | --- | --- | --- | --- | --- | --- | --- | --- | --- | --- |
| **Discovery Set** | Site- I | ATB | NA11 | 23 | F | 137 | 40 | 21.31 | Y | Y | N | N | Y | N | N | Scanty | Y | | S | +ve | ATB |  |  | | | |
|  |  |  | NA12 | 27 | M | 155 | 49 | 20.40 | Y | Y | N | Y | Y | Y | N | 3+ | Y | | S | +ve | ATB |  |  | | | |
|  |  |  | NA13 | 35 | M | 170 | 38 | 13.15 | Y | Y | N | Y | Y | Y | N | 1+ | Y | | S | +ve | ATB |  |  | | | |
|  |  |  | NA14 | 40 | M | 165 | 57 | 20.94 | Y | Y | N | N | Y | Y | N | 1+ | Y | | M | +ve | ATB |  |  | | | |
|  |  |  | NA15 | 36 | M | 171 | 70 | 23.94 | Y | Y | Y | N | Y | Y | Y | 2+ | Y | | M | +ve | ATB |  |  | | | |
|  |  |  | NA16 | 34 | M | 153 | 42 | 17.94 | Y | Y | Y | N | N | Y | N | 3+ | Y | | M | +ve | ATB |  |  | | | |
|  |  |  | NA17 | 58 | M | 159 | 50 | 19.78 | Y | Y | Y | N | Y | Y | N | 2+ | Y | | M | +ve | ATB |  |  | | | |
|  |  |  | NA18 | 37 | M | 157 | 48 | 19.47 | Y | Y | N | Y | Y | Y | Y | Scanty | Y | | M | +ve | ATB |  |  | | | |
|  |  |  | NA19 | 47 | M | 149 | 47 | 21.17 | Y | Y | N | N | Y | Y | Y | 1+ | Y | | S | +ve | ATB |  |  | | | |
|  |  |  | NA20 | 49 | M | 150 | 45 | 20.00 | Y | Y | N | N | Y | Y | Y | 1+ | Y | | S | +ve | ATB |  |  | | | |
|  |  | NTB | NS11 | 35 | M | 165 | 58 | 21.30 | Y | Y | N | N | Y | Y | N | -ve | NA | | NA | -ve | NTB |  |  | | | |
|  |  |  | NS12 | 32 | F | 146 | 39 | 18.30 | Y | Y | N | N | N | N | N | -ve | NA | | NA | -ve | NTB |  |  | | | |
|  |  |  | NS13 | 47 | F | 152 | 40 | 17.31 | Y | Y | N | N | N | N | N | -ve | NA | | NA | -ve | NTB |  |  | | | |
|  |  |  | NS14 | 48 | F | 158 | 49 | 19.63 | Y | Y | Y | N | Y | N | N | -ve | NA | | NA | -ve | NTB |  |  | | | |
|  |  |  | NS15 | 35 | M | 171 | 52 | 17.78 | Y | Y | N | N | Y | Y | Y | -ve | NA | | NA | -ve | NTB |  |  | | | |
|  |  |  | NS16 | 50 | F | 151 | 51 | 22.37 | Y | Y | Y | N | N | N | N | -ve | N | | NA | -ve | NTB |  |  | | | |
|  |  |  | NS17 | 22 | F | 140 | 44 | 22.45 | Y | Y | Y | N | N | N | N | -ve | NA | | NA | -ve | NTB |  |  | | | |
|  |  |  | NS18 | 27 | F | 155 | 56 | 23.31 | Y | Y | N | N | N | N | N | -ve | NA | | NA | -ve | NTB |  |  | | | |
|  |  |  | NS19 | 19 | M | 177 | 41 | 13.09 | Y | Y | N | Y | Y | N | N | -ve | Y | | PE | -ve | NTB |  |  | | | |
|  |  |  | NS20 | 40 | M | 155 | 42 | 17.48 | Y | Y | N | Y | Y | N | N | -ve | NA | | NA | -ve | NTB |  |  | | | |
| **Validation Set** | Site- I | ATB | NA01 | 55 | F | 148 | 50 | 22.83 | Y | Y | Y | Y | Y | N | N | 3+ | Y | | Y | +ve | ATB |  |  | | | |
|  |  |  | NA02 | 28 | F | 150 | 49 | 21.78 | Y | Y | Y | Y | Y | N | N | 3+ | Y | | Y | +ve | ATB |  |  | | | |
|  |  |  | NA03 | 65 | M | 171 | 50 | 17.10 | Y | Y | Y | Y | Y | Y | N | 3+ | Y | | Y | +ve | ATB |  |  | | | |
|  |  |  | NA04 | 23 | M | 146 | 39 | 18.30 | Y | Y | Y | Y | Y | N | N | 1+ | Y | | Y | +ve | ATB |  |  | | | |
|  |  | NTB | NS01 | 59 | F | 157 | 52 | 21.10 | Y | Y | Y | Y | Y | N | N | -ve | NA | | NA | -ve | NTB |  |  | | | |
|  |  |  | NS02 | 20 | F | 146 | 38 | 17.83 | Y | Y | Y | Y | Y | N | N | -ve | N | | N | -ve | NTB |  |  | | | |
|  |  |  | NS03 | 52 | M | 155 | 50 | 20.81 | Y | Y | Y | Y | Y | Y | N | -ve | NA | | NA | -ve | NTB |  |  | | | |
|  |  |  | NS04 | 30 | M | 174 | 58 | 19.16 | Y | Y | Y | Y | N | N | N | -ve | NA | | NA | -ve | NTB |  |  | | | |
|  | Site- II | ATB | RA1 | 60 | F | 155 | 42 | 17.48 | Y | Y | Y | Y | Y | N | N | 2+ | Y | | Y | +ve | ATB |  |  | | | |
|  |  |  | RA2 | 32 | M | 173 | 60 | 20.05 | Y | Y | Y | Y | Y | N | Y | +ve | Y | | Y | +ve | ATB |  |  | | | |
|  |  |  | RA3 | 62 | M | NA | 60 | - | Y | Y | Y | Y | Y | Y | Y | +ve | Y | | Y | +ve | ATB |  |  | | | |
|  |  |  | RA7 | 42 | F | NA | 36 | - | Y | Y | Y | Y | Y | Y | Y | 2+ | Y | | Y | +ve | ATB |  |  | | | |
|  |  |  | RA16 | 40 | F | NA | 46 | - | Y | N | Y | N | Y | N | N | +ve | Y | | Y | +ve | ATB |  |  | | | |
|  |  |  | RA12 | 44 | M | 170 | 42 | 14.53 | Y | Y | Y | Y | Y | Y | Y | +ve | N | | N | +ve | ATB |  |  | | | |
|  |  | NTB | RS1 | 72 | F | NA | 50 | - | Y | Y | Y | Y | Y | N | N | -ve | Y | | Y | -ve | NTB |  |  | | | |
|  |  |  | RS2 | 33 | F | 152 | 75 | 32.46 | Y | Y | Y | Y | Y | Y | N | -ve | Y | | Y | -ve | NTB |  |  | | | |
|  |  |  | RS3 | 63 | M | NA | 40 | - | Y | Y | Y | Y | Y | N | Y | -ve | NA | | NA | -ve | NTB |  |  | | | |
|  |  |  | RS11 | 26 | M | NA | 48 | - | Y | Y | Y | Y | N | N | Y | -ve | N | | N | -ve | NTB |  |  | | | |
|  |  |  | RS12 | 47 | M | 170 | 55 | 19.03 | Y | N | Y | N | Y | N | N | -ve | Y | | Y | -ve | NTB |  |  | | | |
|  |  |  | RS13 | 70 | F | NA | 40 | - | Y | Y | Y | N | Y | Y | Y | -ve | Y | | Y | -ve | NTB |  |  | | | |
|  | Site- III | ATB | NH18 | 45 | M | 175 | 53 | 17.31 | Y | Y | Y | N | NA | Y | Y | +ve | Y | | Y | +ve | ATB |  |  | | | |
|  |  |  | NH20 | 79 | M | 170 | 62 | 21.45 | Y | Y | Y | N | NA | Y | Y | +ve | NA | | NA | +ve | ATB |  |  | | | |
|  |  |  | NH34 | 17 | F | NA | 40 | - | Y | Y | Y | Y | NA | N | Y | +ve | NA | | NA | +ve | ATB |  |  | | | |
|  |  |  | NH40 | 50 | F | 157 | 40 | 16.23 | Y | Y | Y | Y | NA | N | N | +ve | NA | | NA | +ve | ATB |  |  | | | |
|  |  | NTB | NH 12 | 22 | M | 165 | 52 | 19.10 | Y | Y | Y | Y | NA | N | N | -ve | Y | | Y | -ve | NTB |  |  | | | |
|  |  |  | NH43 | 30 | F | 146 | 42 | 19.70 | Y | Y | Y | Y | NA | N | N | -ve | Y | | Y | -ve | NTB |  |  | | | |
|  |  |  | NH49 | 21 | F | NA | NA | - | Y | Y | Y | N | NA | Y | Y | -ve | N | | N | -ve | NTB |  |  | | | |
|  |  |  | NH06 | 32 | M | 174 | 66 | 21.80 | Y | Y | Y | Y | NA | Y | Y | -ve | N | | N | -ve | NTB |  |  | | | |
|  | Site- IV | ATB | AMC09 | 52 | M | 150 | 33 | 14.67 | Y | Y | Y | Y | N | NA | NA | 3+ | Y | | Y | +ve | ATB |  |  | | | |
|  |  |  | AMC17 | 43 | M | 159 | 49 | 19.38 | Y | Y | Y | Y | N | NA | NA | 3+ | Y | | Y | +ve | ATB |  |  | | | |
|  |  |  | AMC22 | 18 | M | 152 | 46 | 19.91 | Y | Y | Y | Y | N | NA | NA | 1+ | Y | | Y | +ve | ATB |  |  | | | |
|  |  |  | AMC23 | 25 | M | 153 | 42 | 17.94 | Y | Y | Y | Y | N | NA | NA | 2+ | NA | | NA | +ve | ATB |  |  | | | |
|  |  | NTB | AMC48 | 61 | F | 162 | 42 | 16.00 | Y | Y | Y | Y | N | NA | NA | -ve | NA | | NA | -ve | NTB |  |  | | | |
|  |  |  | AMC52 | 16 | F | 143 | 31 | 15.16 | Y | Y | Y | Y | N | NA | NA | -ve | NA | | NA | -ve | NTB |  |  | | | |
|  |  |  | AMC53 | 70 | M | 169 | 55 | 19.26 | Y | Y | Y | Y | N | NA | NA | -ve | NA | | NA | -ve | NTB |  |  | | | |
|  |  |  | AMC58 | 66 | M | 150 | 45 | 20.00 | Y | Y | Y | Y | N | NA | NA | -ve | NA | | NA | -ve | NTB |  |  | | | |
| **Follow up Set** | Site- I | Responder ATB | KJ0P010 | 30 | M | 155 | 43 | 17.90 | Y | Y | Y | N | Y | Y | N | 1+ | Y | | Y | +ve | ATB | -ve |  | | | |
|  |  |  | KC0P009 | 25 | M | 142 | 42 | 20.83 | Y | Y | Y | N | N | Y | Y | Scanty | Y | | Y | +ve | ATB | -ve |  | | | |
|  |  |  | KC0P013 | 35 | F | 154 | 38 | 16.02 | Y | Y | Y | N | N | Y | N | 3+ | Y | | Y | +ve | ATB | -ve |  | | | |
|  |  |  | KC0P014 | 23 | M | 144 | 44 | 21.22 | Y | Y | Y | Y | Y | N | N | 3+ | Y | | Y | +ve | ATB | -ve |  | | | |
|  |  | Non-responder ATB | KC0P016 | 38 | F | 154 | 42 | 17.71 | Y | Y | Y | Y | Y | N | N | 1+ | Y | | Y | +ve | ATB | Sc |  | | | |
|  |  |  | KC0P018 | 40 | M | 129 | 38 | 22.84 | Y | Y | Y | Y | Y | Y | N | 1+ | Y | | Y | +ve | ATB | Sc |  | | | |
|  |  |  | KC0P004 | 49 | M | 167 | 28 | 10.04 | Y | Y | Y | Y | Y | N | N | 1+ | Y | | Y | +ve | ATB | 1+ |  | | | |
|  |  |  | KC0P006 | 60 | M | 167 | 57 | 20.44 | Y | Y | Y | Y | Y | Y | Y | 1+ | Y | | Y | +ve | ATB | 1+ |  | | | |
| **Confimatory mixed cohort** | Site- I, II, III, IV,V | ATB and NTB | SP01 | 81 | F | NA | 35 | - | Y | Y | Y | NA | NA | Y | NA | NA | N | | N | -ve | NTB |  |  | | | |
|  |  |  | SP02 | 58 | M | 170 | 60 | 20.76 | Y | Y | Y | N | NA | Y | NA | +ve | Y | | Y | +ve | ATB |  |  | | | |
|  |  |  | SP03 | 65 | M | NA | 59 | - | Y | Y | Y | NA | NA | NA | NA | NA | NA | | NA | -ve | NTB |  |  | | | |
|  |  |  | SP04 | 76 | M | NA | 60 | - | Y | Y | Y | NA | NA | NA | NA | NA | NA | | NA | -ve | NTB |  |  | | | |
|  |  |  | SP05 | 29 | M | 152 | 49 | 21.10 | Y | Y | Y | Y | Y | N | N | +ve | Y | | Y | +ve | ATB |  |  | | | |
|  |  |  | SP06 | 76 | M | NA | 60 | - | Y | Y | Y | NA | NA | NA | NA | NA | NA | | NA | -ve | NTB |  |  | | | |
|  |  |  | SP06 | 72 | M | 170 | 57 | 19.68 | Y | Y | Y | N | Y | Y | N | +ve | NA | | NA | +ve | ATB |  |  | | | |
|  |  |  | SP07 | 40 | M | 163 | 57 | 21.57 | Y | Y | Y | N | Y | N | N | +ve | Y | | Y | +ve | ATB |  |  | | | |
|  |  |  | SP08 | 56 | M | NA | NA | - | Y | Y | Y | NA | NA | Y | NA | +ve | NA | | NA | +ve | ATB |  |  | | | |
|  |  |  | SP09 | 65 | M | 153 | 33 | 14.10 | Y | Y | Y | Y | Y | N | N | -ve | Y | | Y | -ve | NTB |  |  | | | |
|  |  |  | SP10 | 80 | M | NA | 50 | - | Y | Y | Y | N | NA | N | NA | +ve | Y | | Y | +ve | ATB |  |  | | | |
|  |  |  | SP11 | 32 | M | 173 | 60 | 20.05 | Y | Y | Y | Y | NA | N | NA | +ve | Y | | Y | +ve | ATB |  |  | | | |
|  |  |  | SP12 | 26 | M | 173 | 60 | 20.11 | Y | Y | Y | N | Y | N | N | +ve | Y | | Y | +ve | ATB |  |  | | | |
|  |  |  | SP13 | 60 | F | 155 | 42 | 17.48 | Y | Y | Y | N | NA | N | NA | +ve | Y | | Y | +ve | ATB |  |  | | | |
|  |  |  | SP14 | 55 | M | NA | 44 | - | Y | Y | Y | Y | NA | Y | NA | +ve | Y | | Y | +ve | ATB |  |  | | | |
|  |  |  | SP15 | 45 | M | NA | 51 | - | Y | Y | Y | Y | NA | N | NA | NA | Y | | Y | -ve | NTB |  |  | | | |
|  |  |  | SP16 | 17 | F | 135 | 60 | 33.11 | Y | Y | Y | N | Y | N | N | +ve | NA | | NA | +ve | ATB |  |  | | | |
|  |  |  | SP17 | 42 | M | 163 | 70 | 26.49 | Y | Y | Y | Y | Y | N | N | +ve | Y | | Y | +ve | ATB |  |  | | | |
|  |  |  | SP18 | 48 | F | 157 | 49 | 19.76 | Y | Y | Y | N | Y | N | N | -ve | NA | | NA | -ve | NTB |  |  | | | |
|  |  |  | SP19 | 22 | F | 157 | 45 | 18.15 | Y | Y | Y | N | Y | N | N | -ve | NA | | NA | +ve | ATB |  |  | | | |
|  |  |  | SP20 | 27 | M | 145 | 39 | 18.61 | Y | Y | Y | Y | Y | N | N | +ve | NA | | NA | +ve | ATB |  |  | | | |
|  |  |  | SP21 | 48 | M | 155 | 48 | 19.98 | Y | Y | Y | N | Y | N | N | +ve | Y | | Y | +ve | ATB |  |  | | | |
|  |  |  | SP22 | 40 | M | 155 | 42 | 17.50 | Y | Y | Y | Y | Y | N | N | -ve | NA | | NA | -ve | NTB |  |  | | | |
|  |  |  | SP23 | 75 | M | NA | 57 | - | Y | Y | Y | N | NA | Y | NA | -ve | N | | N | -ve | NTB |  |  | | | |
|  |  |  | SP24 | 45 | M | NA | 55 | - | Y | Y | Y | Y | NA | NA | NA | -ve | Y | | NA | -ve | NTB |  |  | | | |
|  |  |  | SP25 | 45 | M | 155 | 36 | 15.00 | Y | Y | Y | Y | N | N | N | +ve | Y | | Y | +ve | ATB |  |  | | | |
|  |  |  | SP26 | 75 | M | NA | 57 | - | Y | Y | Y | NA | NA | NA | NA | NA | N | | N | -ve | NTB |  |  | | | |
|  |  |  | SP27 | 65 | M | 159 | 45 | 17.80 | Y | Y | Y | Y | Y | N | N | -ve | Y | | Y | +ve | ATB |  |  | | | |
|  |  |  | SP28 | 27 | M | 155 | 49 | 20.41 | Y | Y | Y | Y | Y | N | N | +ve | Y | | Y | +ve | ATB |  |  | | | |
|  |  |  | SP29 | 53 | M | 155 | 42 | 17.50 | Y | Y | Y | N | Y | Y | N | +ve | Y | | Y | +ve | ATB |  |  | | | |
|  |  |  | SP30 | 76 | F | NA | 49 | - | Y | Y | Y | NA | NA | NA | NA | NA | NA | | NA | -ve | NTB |  |  | | | |
|  |  |  | SP31 | 55 | F | 147 | 50 | 23.04 | Y | Y | Y | Y | Y | N | N | +ve | Y | | Y | +ve | ATB |  |  | | | |
|  |  |  | SP32 | 40 | F | NA | 46 | - | Y | Y | Y | N | NA | N | NA | +ve | Y | | Y | +ve | ATB |  |  | | | |
|  |  |  | SP33 | 52 | M | NA | 55 | - | Y | Y | Y | Y | NA | NA | NA | -ve | N | | N | -ve | NTB |  |  | | | |
|  |  |  | SP34 | 65 | M | 163 | 40 | 15.14 | Y | Y | Y | N | Y | N | Y | +ve | Y | | Y | +ve | ATB |  |  | | | |
|  |  |  | SP35 | 38 | F | NA | 45 | - | Y | Y | Y | NA | NA | NA | NA | NA | N | | N | -ve | NTB |  |  | | | |
|  |  |  | SP37 | 86 | M | NA | NA | - | Y | Y | Y | N | NA | NA | NA | -ve | N | | N | -ve | NTB |  |  | | | |
|  |  |  | SP38 | 70 | M | 163 | 38 | 14.30 | Y | Y | Y | Y | Y | N | N | -ve | Y | | Y | -ve | NTB |  |  | | | |
|  |  |  | SP39 | 56 | M | 168 | 35 | 12.45 | Y | Y | Y | Y | N | N | N | -ve | NA | | NA | -ve | NTB |  |  | | | |
|  |  |  | SP40 | 32 | F | 142 | 39 | 19.28 | Y | Y | Y | N | N | N | N | -ve | NA | | NA | -ve | NTB |  |  | | | |
|  |  |  | SP41 | 18 | F | 150 | 43 | 19.15 | Y | Y | Y | Y | Y | N | N | -ve | NA | | NA | -ve | NTB |  |  | | | |
|  |  |  | SP41 | 58 | M | 167 | 54 | 19.36 | Y | Y | Y | N | NA | Y | NA | +ve | Y | | Y | +ve | ATB |  |  | | | |
|  |  |  | SP43 | 23 | F | 150 | 33 | 14.69 | Y | Y | Y | N | Y | N | N | +ve | Y | | Y | +ve | ATB |  |  | | | |
|  |  |  | SP44 | 22 | M | 159 | 51 | 20.17 | Y | Y | Y | Y | Y | N | N | +ve | Y | | Y | +ve | ATB |  |  | | | |
|  |  |  | SP45 | 75 | M | NA | 45 | - | Y | Y | Y | N | NA | Y | NA | -ve | Y | | Y | -ve | NTB |  |  | | | |
|  |  |  | SP46 | 50 | F | NA | 54 | - | Y | Y | Y | N | NA | N | NA | NA | N | | N | -ve | NTB |  |  | | | |
|  |  |  | SP47 | 45 | M | 152 | 43 | 18.51 | Y | Y | Y | N | Y | N | N | +ve | Y | | Y | +ve | ATB |  |  | | | |
|  |  |  | SP48 | 38 | M | NA | 55 | - | Y | Y | Y | NA | NA | Y | NA | NA | N | | N | -ve | NTB |  |  | | | |
|  |  |  | SP49 | 24 | M | 172 | 44 | 14.75 | Y | Y | Y | N | Y | N | N | -ve | Y | | Y | -ve | NTB |  |  | | | |
|  |  |  | SP50 | 30 | M | 170 | 58 | 20.03 | Y | Y | Y | N | N | N | N | -ve | NA | | NA | -ve | NTB |  |  | | | |
|  |  |  |  |  |  |  |  |  |  |  |  |  |  |  |  |  |  | |  |  |  |  |  | | | |
|  |  |  |  |  |  |  |  |  |  |  |  |  |  |  |  |  | |  |  |  |  |  | |  | |  |

Y: Yes; N: No; M: male; F: Female; NA: not available; +ve: postitive; -ve: negative;

**Table S-2: List of identified sputum proteins in the iTRAQ experiment (Page S-32 to S-34).**

| 78 kDa glucose-regulated protein | Isoform H7 of Myeloperoxidase |  |
| --- | --- | --- |
| Actin, cytoplasmic 2 | Keratin |  |
| Adenylyl cyclase-associated protein 1 | Keratin 13 |  |
| Alpha-1-antitrypsin | Keratin, type I cytoskeletal 14 |  |
| Alpha-1B-glycoprotein | Keratin, type I cytoskeletal 16 |  |
| Alpha-2-macroglobulin | Keratin, type II cytoskeletal 5 |  |
| Alpha-actinin-1 | Lactoferrin |  |
| Alpha-amylase 1 | Lactoperoxidase |  |
| Alpha-enolase | Lamin-B1 |  |
| ALX5 protein | Leucine-rich alpha-2-glycoprotein |  |
| Annexin A3 | Lipocalin-1 |  |
| Annexin A5 | L-lactate dehydrogenase |  |
| annexin A6 | Long palate, lung and nasal epithelium carcinoma-associated protein 1 |  |
| Antileukoproteinase | Low affinity immunoglobulin gamma Fc region receptor III-A |  |
| Anti-streptococcal/anti-myosin immunoglobulin kappa light chain variable region (Fragment) | Lysozyme C |  |
| Anti-TN-C scFv (Fragment) | matrix metallopeptidase 8 |  |
| ANXA1 protein | Matrix metalloproteinase-9 |  |
| ANXA4 protein (Fragment) | Moesin |  |
| Arginase-1 | Mucin-5AC (Fragments) |  |
| Azurocidin | Mucin-5B |  |
| Bactericidal permeability-increasing protein | Myeloblastin |  |
| Bactericidal/permeability-increasing protein-like 1 | MYH9 variant protein |  |
| Basic salivary proline-rich protein 2 | NCF4 protein |  |
| Bone marrow stromal cell antigen 1 nirs variant 1 | Neutrophil elastase |  |
| Calreticulin variant (Fragment) | Neutrophil gelatinase-associated lipocalin |  |
| Carbonic anhydrase VI nirs variant 1 | Non-secretory ribonuclease |  |
| Catalase | ORM2 protein |  |
| Cathelicidin antimicrobial peptide | Orosomucoid 1 |  |
| Cathepsin G | Palate lung and nasal epithelium carcinoma associated protein |  |
| cDNA FLJ25678 fis, clone TST04067, highly similar to PURINE NUCLE | Pancreatic adenocarcinoma upregulated factor |  |
| cDNA FLJ38670 fis, clone HSYRA2000190, highly similar to Voltage-dependent anion-selective channel protein 1 | Peptidoglycan recognition protein 1 |  |
| cDNA FLJ51518, highly similar to Annexin A11 | Peptidyl-prolyl cis-trans isomerase |  |
| cDNA FLJ55574, highly similar to Calnexin | Peptidyl-prolyl cis-trans isomerase B |  |
| cDNA FLJ57067, highly similar to Beta-2-microglobulin | Phosphoglycerate mutase (Fragment) |  |
| cDNA FLJ75641, highly similar to Homo sapiens v-yes-1 Yamaguchi sarcoma viral related oncogene homolog (LYN), mRNA | Phospholipase B-like 1 |  |
| ceruloplasmin (ferroxidase) | Plasma protease C1 inhibitor |  |
| Chromosome 20 open reading frame 3 (Fragment) | Plastin-2 |  |
| Clusterin | Polymeric immunoglobulin receptor |  |
| CNN2 protein | Polytrophin |  |
| Collagen, type VI, alpha 3 | Profilin |  |
| Complement C3 | Prolactin-inducible protein |  |
| Complement C4-B | Proline-rich protein 4 |  |
| Copine-3 | Protein disulfide-isomerase |  |
| Coronin-1A | Protein S100-A11 |  |
| CSTB protein | Protein S100-A12 |  |
| Cystatin-S | Protein S100-A4 |  |
| Cystatin-SA | Protein S100-A6 |  |
| Cystatin-SN | Protein S100-A7 |  |
| Cysteine-rich secretory protein 3 | Protein S100-A8 |  |
| Cytochrome b-245 heavy chain | Protein S100-A9 |  |
| Defensin, alpha 1 | Protein S100-A9 |  |
| Deleted in malignant brain tumors 1 protein | Protein S100-P |  |
| Delta globin | PSME2 protein |  |
| DKFZp686I04196 | Putative uncharacterized protein |  |
| DKFZp686L19235 | Putative uncharacterized protein |  |
| DKFZp686P15220 | Pyruvate kinase isozymes M1/M2 |  |
| EEF1G protein | Resistin |  |
| Eosinophil cationic protein | Rho GDP-dissociation inhibitor 2 |  |
| Eosinophil peroxidase | Salivary acidic proline-rich phosphoprotein 1/2 |  |
| Erythrocyte band 7 integral membrane protein | SCCA1/SCCA2 fusion protein |  |
| Ezrin | Serum albumin |  |
| Fatty acid binding protein 5 (Psoriasis-associated) | SNC73 protein |  |
| FGA protein (Fragment) | Spectrin beta chain, brain 4 |  |
| Fibrinogen beta chain | Superoxide dismutase (Fragment) |  |
| Filamin A, alpha (Actin binding protein 280) | Tetraspanin-1 |  |
| Galectin-3-binding protein | Thioredoxin |  |
| GDI2 protein | Thymosin beta-4-like protein 3 |  |
| Gelsolin | TIMP metallopeptidase inhibitor 1 |  |
| Glucose-6-phosphate isomerase | Transaldolase |  |
| Glyceraldehyde-3-phosphate dehydrogenase | Transcobalamin-1 |  |
| Haptoglobin | Transferrin variant (Fragment) |  |
| Heat shock 70kDa protein 1A variant (Fragment) | Transketolase variant (Fragment) |  |
| Heat shock 70kDa protein 8 isoform 1 variant (Fragment) | Triosephosphate isomerase |  |
| Hemoglobin, beta | Tubulin, alpha 1 (Testis specific), isoform CRA_a |  |
| Hemopexin | Tubulin, beta 2C |  |
| Heterogeneous nuclear ribonucleoprotein A1 | UBC protein (Fragment) |  |
| Histone H2A type 2-A | Uncharacterized protein |  |
| Histone H2A type 2-B | Uncharacterized protein |  |
| Histone H2B | Uncharacterized protein |  |
| Histone H2B type 1-J | Uncharacterized protein |  |
| Histone H4 | Uncharacterized protein |  |
| Ig gamma-4 chain C region | Uncharacterized protein |  |
| Ig heavy chain V-I region V35 | Uncharacterized protein |  |
| Ig kappa chain V-II region GM607 (Fragment) | Uncharacterized protein |  |
| Ig kappa chain V-IV region Len | UPF0762 protein C6orf58 |  |
| Ig lambda chain V-I region NEW | Uteroglobin |  |
| Ig mu chain C region | Vasodilator-stimulated phosphoprotein |  |
| IgGFc-binding protein | Vimentin variant 3 |  |
| IGK@ protein | Vinculin |  |
| IGL@ protein | Vitamin D-binding protein |  |
| IGL@ protein | Vitronectin |  |
| Immunglobulin heavy chain variable region (Fragment) | Zinc-alpha-2-glycoprotein |  |
| Immunglobulin heavy chain variable region (Fragment) |  |  |
| Immunoglobulin J chain |  |  |
| Integrin alpha-M |  |  |
| Integrin beta (Fragment) |  |  |
| IQ motif containing GTPase activating protein 1 |  |  |
| Isoform 2 of Annexin A2 |  |  |
| Isoform 2 of Brain acid soluble protein 1 |  |  |
| Isoform 2 of Kallikrein-1 |  |  |
